# Gli3 in fetal thymic epithelial cells promotes thymocyte positive selection and differentiation by repression of *Shh*

**DOI:** 10.1242/dev.146910

**Published:** 2018-02-01

**Authors:** Anisha Solanki, Diana C. Yanez, Susan Ross, Ching-In Lau, Eleftheria Papaioannou, Jiawei Li, José Ignacio Saldaña, Tessa Crompton

**Affiliations:** 1 UCL GOS Institute of Child Health, 30 Guilford Street, London WC1N 1EH, UK; 2 School of Health, Sport and Bioscience, University of East London, London E15 4LZ, UK

**Keywords:** Shh, Gli3, Fetal thymus, Positive selection, CD4, T-cell development, Thymocyte, Thymic epithelial cell (TEC), Mouse

## Abstract

Gli3 is a Hedgehog (Hh)-responsive transcription factor that can function as a transcriptional repressor or activator. We show that Gli3 activity in mouse thymic epithelial cells (TECs) promotes positive selection and differentiation from CD4^+^ CD8^+^ to CD4^+^ CD8^−^ single-positive (SP4) cells in the fetal thymus and that Gli3 represses *Shh*. Constitutive deletion of *Gli3*, and conditional deletion of *Gli3* from TECs, reduced differentiation to SP4, whereas conditional deletion of *Gli3* from thymocytes did not. Conditional deletion of *Shh* from TECs increased differentiation to SP4, and expression of Shh was upregulated in the Gli3-deficient thymus. Use of a transgenic Hh reporter showed that the Hh pathway was active in thymocytes, and increased in the Gli3-deficient fetal thymus. Neutralisation of endogenous Hh proteins in the *Gli3*^−/−^ thymus restored SP4 differentiation, indicating that Gli3 in TECs promotes SP4 differentiation by repression of *Shh*. Transcriptome analysis showed that Hh-mediated transcription was increased whereas TCR-mediated transcription was decreased in *Gli3*^−/−^ thymocytes compared with wild type.

## Abstract (precis)

**Summary:** Gli3 activity in thymic epithelial cells promotes maturation from CD4^+^ CD8^+^ to CD4^+^ CD8^−^ thymocyte by reducing Shh, which signals to reduce T-cell receptor-mediated transcription during repertoire selection in mouse fetal thymus.

## INTRODUCTION

Gli3 is a member of the Hedgehog (Hh)-responsive Gli family of transcription factors, mammalian orthologues of the *Drosophila* Ci protein (Ramsbottom and Pownall, 2016). The Gli proteins bind DNA in a sequence-specific manner, but have evolved different functions and distinct temporal and tissue-specific expression patterns. Gli3 can be processed to be a repressor of transcription (Gli3R) in the absence of Hh signalling, or an activator (Gli3A) upon Hh signal transduction (Sasaki et al., 1999). During development it can function before the expression of *Hh* genes, independently of Hh. In many tissues, Gli3R limits Shh signalling, Gli3R and Shh have opposing functions, and Gli3 deficiency and Shh deficiency result in opposite phenotypes (Hager-Theodorides et al., 2005; Shah et al., 2004; Solanki et al., 2017; te Welscher et al., 2002; Wang et al., 2000).

During αβ T-cell development in the thymus, CD4^−^ CD8^−^ double-negative (DN) cells differentiate to CD4^+^ CD8^+^ double-positive (DP) cells, which give rise to both CD4 single-positive (SP4) and CD8 single-positive (SP8) populations. Gli3 is expressed in adult and fetal thymic epithelial cells (TECs) and fetal but not adult thymocytes, and Gli3 promotes pre-T-cell receptor (TCR)-induced differentiation from DN to DP cell, and negative selection of the TCR repertoire (Barbarulo et al., 2016; Hager-Theodorides et al., 2005, 2009; Saldaña et al., 2016). Here, we investigate Gli3 function during αβ T-cell development in the embryonic thymus at the transition from the DP to SP cell.

Maturation from DP to SP follows successful rearrangement of the *Tcra* locus, and requires TCR signalling: positive selection results in appropriate MHC restriction of SP cells, followed by negative selection of potentially self-reactive clones (Klein et al., 2014; Starr et al., 2003). Many models have been proposed to describe how DP thymocytes commit to the SP4 and SP8 lineages, and how positive selection ensures that selected SP4 and SP8 populations express TCR appropriately restricted by MHCII and MHCI, respectively (Carpenter and Bosselut, 2010; Starr et al., 2003). The strength and duration of the TCR signal that a developing cell receives broadly determine its fate, with the strongest signals leading to negative selection, usually at the SP stage in the medulla (of TCR recognising self antigens), intermediate signals leading to positive selection, and weaker signals or lack of TCR signalling leading to cell death by neglect (Singer et al., 2008). For DP thymocytes undergoing positive selection, again TCR signal strength and duration influence SP4 and SP8 lineage choice. Those cells receiving stronger longer TCR signals tend towards the SP4 fate, weaker/more transient signals favour differentiation to SP8 SP, and additionally SP4/SP8 fate decisions may be influenced by the relative timing of cytokine signalling and TCR signalling that a developing cell receives (Bosselut, 2004; Klein et al., 2014; Starr et al., 2003). TCR signal strength and duration are dependent on avidity of the TCR for its ligand (and therefore on the TCR sequence), and may also be affected by other intracellular or extracellular influences on TCR signal transduction, in addition to cytokines. Thus, local thymic stromal factors, including Notch and morphogen signalling, may also influence SP lineage choice and selection (Brugnera et al., 2000; Crompton et al., 2007; Laky and Fowlkes, 2008; Park et al., 2010; Takahama, 2006). Several lineage-specific transcription factors are required for the SP4/SP8 lineage decision, including ThPok (Zbtb7b), Gata3, Runx1, Runx3 and Mazr (Carpenter and Bosselut, 2010; Naito et al., 2011). The ways in which the transcriptional regulation of lineage commitment and differentiation relate to extracellular signalling molecules and TCR signal transduction require further study.

In the thymus, Shh is expressed by TECs in the medulla and corticomedullary junction, and is required for normal medullary TEC development and maturation (El Andaloussi et al., 2006; Outram et al., 2000; Sacedón et al., 2003; Saldaña et al., 2016). TECs provide MHCpeptide ligands for developing thymocytes and are required for both positive and negative selection of the TCR repertoire (Klein et al., 2014).

Gli3R can suppress Hh pathway activation by at least two mechanisms. First, it can repress the expression of *Hh* genes in the Hh-secreting cell, hence reducing the overall Hh protein concentration in a tissue. Second, expression of Gli3 in the signal-receiving cell will be processed to Gli3R in the absence of Hh proteins, which will transcriptionally repress Hh target genes.

In the thymus stroma, Gli3 has both Hh-independent and Hh-dependent functions, and Gli3 deficiency leads to Hh-dependent upregulation of the Hh target gene *Gli1* (Hager-Theodorides et al., 2009). In the fetal thymus, Gli3 deficiency leads to reduced pre-TCR-induced differentiation from DN to DP, whereas Shh deficiency leads to the opposite phenotype (Hager-Theodorides et al., 2005; Rowbotham et al., 2009). Later in T-cell development, *Gli3* mutation reduces negative selection and influences the transcriptome of fetal thymus stromal cells (Hager-Theodorides et al., 2009). Stromally expressed genes influenced by *Gli3* mutation include Hedgehog-interacting protein (*Hhip*), *Rbp1*, *Cxcl9* and *Nos2*. This suggested that Gli3 might influence negative selection through regulation of *Nos2* (Hager-Theodorides et al., 2009).

Here we show that Gli3 expressed in TECs promotes positive selection and maturation from DP to SP T-cell by suppression of *Shh* expression in TECs, and that Shh signals directly to developing T-cells to modulate TCR-mediated signalling and transcription.

## RESULTS

### Impaired development of mature SP4 T-cells in the *Gli3* mutant thymus

**Fig. 1.**
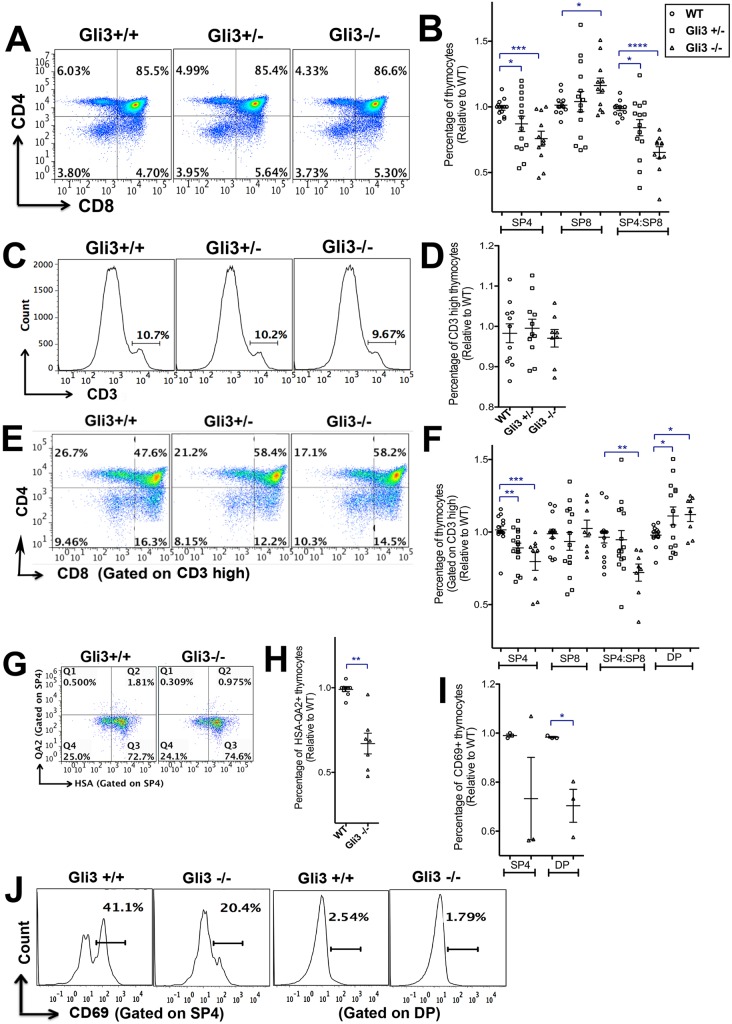
**T-cell development in *Gli3*^+/+^, *Gli3*^+/−^ and *Gli3*^−/−^ E17.5 plus 4 days in FTOC and E18.5 thymus.** Flow cytometry analysis. Scatter plots show mean±s.e.m. Each point represents thymus from an individual mouse embryo. (A-H) E17.5 FTOC for 4 days. *Gli3*^+/+^ (*n*=14), *Gli3*^+/−^ (*n*=14) and *Gli3*^−/−^ (*n*=11). (I,J) E18.5 *Gli3*^+/+^ (*n*=3) and *Gli3*^−/−^ (*n*=3) thymus. (A) CD8 against CD4. (B) Percentage of populations (relative to mean of WT) giving significance by Student's *t*-test compared with WT for SP4 (*Gli3*^+/−^, *P*<0.03; *Gli3*^−/−^, *P*<0.001), SP8 (*Gli3*^−/−^, *P*<0.03) and SP4:SP8 ratio (*Gli3*^+/−^, *P*<0.02; *Gli3*^−/−^, *P*<0.0006). (C) CD3 staining on *Gli3*^+/+^, *Gli3*^+/−^ and *Gli3*^−/−^ thymocytes, giving the percentage of CD3^hi^ cells. (D) Percentage of CD3^hi^ thymocytes in *Gli3*^+/+^, *Gli3*^+/−^ and *Gli3*^−/−^ (relative to mean of WT littermate). (E) CD8 against CD4, gated on CD3^hi^. (F) Percentage of populations gated on CD3^hi^, giving significance by Student's *t*-test compared with WT for CD3^hi^ SP4 (*Gli3*^+/−^, *P*<0.002; *Gli3*^−/−^, *P*<0.0004), CD3^hi^ SP4:SP8 ratio (*Gli3*^−/−^, *P*<0.002) and CD3^hi^ DP (*Gli3*^+/−^, *P*<0.05; *Gli3*^−/−^, *P*<0.02). (G) HSA against Qa2 expression, gated on SP4 cells from *Gli3*^+/+^ and *Gli3*^−/−^. (H) Relative percentage of HSA^−^ Qa2^+^ cells in the SP4 population, giving significance by Student's *t*-test compared with WT (*P*<0.002). (I) Percentage of CD69^+^ cells in SP4 and DP populations, giving significance by Student's *t*-test for SP4 (*P*<0.07) and DP (*P*<0.05). (J) CD69 expression on SP4 and DP cells in a representative experiment.

Gli3 deficiency is embryonic lethal, so to investigate whether Gli3 is required for differentiation of thymocytes from the DP to SP stage, we cultured wild-type (WT) and *Gli3* mutant E17.5 mouse fetal thymus organ culture (FTOC) for 4 days and assessed changes in developmentally regulated cell-surface markers. This culture period enabled us to measure the rate of differentiation of the mature SP populations, as they are first produced. We observed a significant gene dose-dependent decrease in the proportion of SP4 cells and in the SP4:SP8 ratio in the *Gli3* mutant FTOC compared with WT (Fig. 1A,B). The SP4 population was TCRβ^+^ and TCRγδ^−^ in both WT and *Gli3* mutant thymus (Fig. S1), consistent with normal divergence of the γδ lineage at the DN stage in the *Gli3*^−/−^ thymus (Vantourout and Hayday, 2013). The proportion of CD4^−^ CD8^+^ cells was significantly increased in *Gli3*^−/−^ compared with WT (Fig. 1A,B). This suggests that efficient differentiation from DP to SP4 cells required Gli3, and that Gli3 deficiency favoured lineage commitment to SP8 over SP4. However, the embryonic CD8^+^ CD4^−^ population also contains immature single positive (ISP) cells, so we gated on the CD3^hi^ population, and analysed the distribution of DP and SP thymocytes. We found no significant difference in the proportion of CD3^hi^ thymocytes between WT, *Gli3*^+/−^ and *Gli3*^−/−^ (Fig. 1C,D). Gating on CD3^hi^ cells confirmed the requirement for Gli3 for normal differentiation from CD3^hi^ DP to CD3^hi^ SP4 cell, as the proportion of CD3^hi^ DP cells was significantly increased and the proportion of CD3^hi^ SP4 cells was significantly decreased, whereas the proportion of the CD3^hi^ SP8 population was not significantly different between the three genotypes of embryo (Fig. 1E,F).

In order to dissect further the stages of maturity affected by Gli3, we stained thymocytes for the surface markers CD69, HSA (CD24) and Qa2. DP thymocytes express high levels of HSA and then acquire CD69 expression as a result of TCR signalling for positive selection (Ge and Chen, 1999). Newly positively selected SP thymocytes also express high levels of HSA and CD69, and as they mature they downregulate HSA and CD69 and gain expression of Qa2 (Ge and Chen, 1999; Weinreich and Hogquist, 2008).

The proportion of mature HSA^−^ Qa2^+^ cells in the SP4 population, although low in both genotypes, was significantly decreased in *Gli3*^−/−^ compared with WT (Fig. 1G,H). CD69 expression was significantly decreased on DP thymocytes in the *Gli3*^−/−^ thymus compared with WT (Fig. 1I,J), indicating that fewer DP cells were undergoing positive selection, and consistent with the overall reduction in SP4 maturation. CD69 expression was also on average lower on the *Gli3*^−/−^ SP4 population than WT, and there was greater variation in expression levels in *Gli3*^−/−^ compared with WT, suggesting dysregulated maturation in the absence of Gli3.

**Fig. 2.**
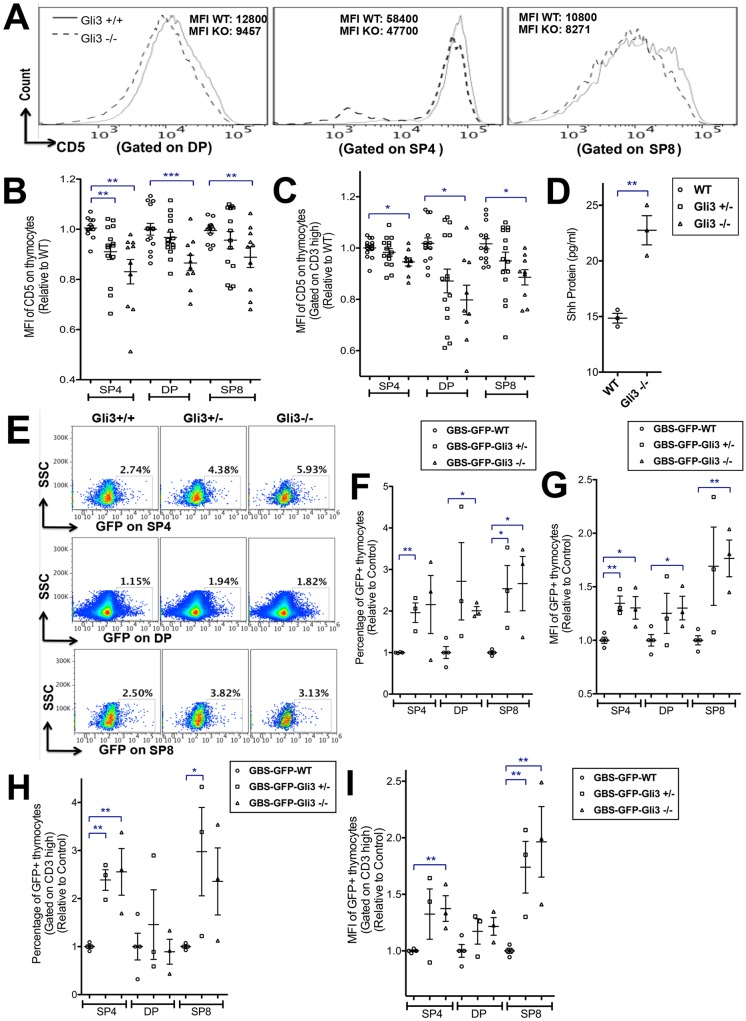
**Expression of CD5 on thymocytes, ELISA for Shh in E17.5 thymus, and detection of Hh pathway activation in E17.5 WT and *Gli3* mutant thymocytes.** (A-C) Flow cytometry analysis of E17.5 FTOC +4 days, with scatter plots showing relative mean±s.e.m., where each point represents thymus from an individual embryo from *Gli3*^+/+^ (WT; *n*=14, circles), *Gli3*^+/−^ (*n*=14, squares), *Gli3*^−/−^ (*n*=11, triangles). (A) CD5 staining on DP, SP4 and SP8 populations, showing MFIs from a representative experiment. (B) MFI of CD5 on SP4, DP and SP8 populations, giving significance by Student's *t*-test compared with WT for SP4 (*Gli3*^+/−^, *P*<0.007; *Gli3*^−/−^, *P*<0.004), DP (*Gli3*^−/−^, *P*<0.001) and SP8 (*Gli3*^−/−^, *P*<0.004). (C) MFI of CD5, gated on CD3^hi^ on SP4, DP and SP8, giving significance by Student's *t*-test for CD3^hi^ SP4 (*Gli3*^−/−^, *P*<0.03), CD3^hi^ DP (*Gli3*^−/−^, *P*<0.04) and CD3^hi^ SP8 (*Gli3*^−/−^, *P*<0.04). (D) Shh protein measured by ELISA in *Gli3*^+/+^ (*n*=3) and *Gli3*^−/−^ (*n*=3) E17.5 thymus (*P*<0.003). (E-I) Flow cytometry analysis of GFP expression in SP4, DP and SP8 populations from GBS-GFP-tg E17.5 FTOC +4 days from *Gli3*^+/+^ (*n*=3), *Gli3*^+/−^ (*n*=4) and *Gli3*^−/−^ (*n*=3) littermates. Scatter plots show relative mean± s.e.m., where each point represents thymus from a different embryo. (E) SSC versus GFP, gated on SP4 (top row), DP (middle row) and SP8 (bottom row) cells from *Gli3*^+/+^ (left), *Gli3*^+/−^ (middle) and *Gli3*^−/−^ (right) littermates, with percentage in region shown. (F) Percentage GFP^+^, giving significance by Student's *t*-test compared with WT for SP4 (*Gli3*^+/−^, *P*<0.004), DP (*Gli3*^−/−^, *P*<0.003) and SP8 (*Gli3*^+/−^, *P*<0.02; *Gli3*^−/−^, *P*<0.02). (G) MFI of GFP, giving significance by Student's *t*-test compared with WT for SP4 (*Gli3*^+/−^, *P*<0.003; *Gli3*^−/−^, *P*<0.02), DP (*Gli3*^−/−^, *P*<0.04) and SP8 (*Gli3*^−/−^, *P*<0.004). (H) Percentage of GFP^+^ cells, gated on CD3^hi^, giving significance by Student's *t*-test compared with WT for CD3^hi^ SP4 (*Gli3*^+/−^, *P*<0.006; *Gli3*^−/−^, *P*<0.01) and CD3^hi^ SP8 (*Gli3*^+/−^, *P*<0.05). (I) MFI of GFP, gated on CD3^hi^, giving significance by Student's *t*-test compared with WT for SP4 (*Gli3*^−/−^, *P*<0.01) and SP8 (*Gli3*^+/−^, *P*<0.01; *Gli3*^−/−^, *P*<0.01).

Positive and negative selection and SP4/8 lineage commitment are determined by many factors, including transcriptional regulators of signal transduction and the TCR signal strength itself. A longer and stronger TCR signal promotes differentiation towards SP4, whereas a weaker signal favours SP8 (Bosselut, 2004). Since Gli3 deficiency suppressed lineage commitment towards SP4 cells and biased the SP4:SP8 ratio, we investigated whether the TCR signal strength was affected in the *Gli3* mutant FTOC by measuring cell-surface CD5 expression, which correlates with TCR and pre-TCR signal strength (Azzam et al., 2001, 1998). The mean fluorescence intensity (MFI) of CD5 on the DP, SP4 and SP8 cells was significantly decreased in the *Gli3*^−/−^ thymus compared with WT (Fig. 2A-C). This suggested that reduced TCR signal strength might be one factor responsible for the decreased commitment to SP4 in the *Gli3* mutant.

### Increased Shh signalling in *Gli3* mutant thymocytes

The *Gli3* mutant fetal thymus has increased expression of the Hh target gene *Gli1* in stroma (Hager-Theodorides et al., 2009), indicating that, overall, Gli3 acts as a repressor of Hh pathway activation in the stroma. Since Gli3 can repress *Shh* expression by repression of an intermediate transcriptional activator of *Shh* in other tissues (te Welscher et al., 2002), and Shh is the key Hh ligand expressed by TECs (Outram et al., 2000; Rowbotham et al., 2007; Saldaña et al., 2016; Shah et al., 2004), we tested whether more Shh protein was present in the Gli3-deficient fetal thymus compared with WT by ELISA. Shh protein was significantly increased in *Gli3*^−/−^ compared with WT (Fig. 2D).

To test if Shh was signalling directly to developing T-cells, we used Gli binding site (GBS)-GFP transgenic (tg) reporter mice to measure active Hh-dependent transcription in DP, SP4 and SP8 populations in the *Gli3* mutants (Fig. 2E-I). The GBS-GFP-tg express GFP when activator forms of Gli proteins bind to the GBS transgene (Balaskas et al., 2012). We observed significant increases in GFP expression in DP, SP4 and SP8/ISP populations in GBS-GFP-tg *Gli3* mutants compared with GBS-GFP-tg *Gli3*^+/+^ (Fig. 2E,F). The MFI of GFP on the SP4, DP and SP8 populations was significantly higher in *Gli3*^−/−^ than in *Gli3*^+/+^ (Fig. 2G). Gating on CD3^hi^ thymocytes, the proportion of GFP^+^ mature CD3^hi^ SP4 cells was significantly higher in the *Gli3* mutants compared with *Gli3*^+/+^, and the MFI of CD3^hi^ SP4 and CD3^hi^ SP8 cells was also significantly increased (Fig. 2H,I). This increase in GFP expression showed that Hh pathway activation is increased in thymocytes in *Gli3*^−/−^ and therefore suggests that the increased Shh protein level is signalling directly to developing T-cells in the *Gli3* mutant thymus.

### Attenuation of Hh signalling in *Gli3*^−/−^ thymus reverses the decrease in the SP4 population

**Fig. 3.**
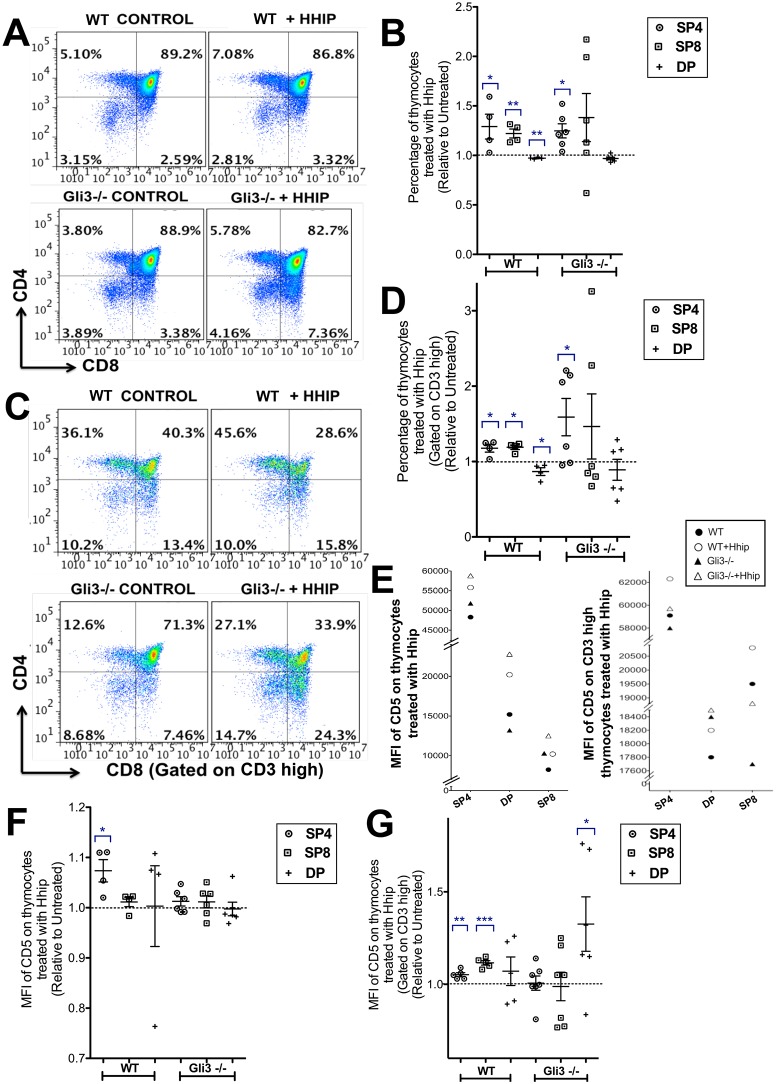
**Attenuation of Hh signalling by rHhip treatment of WT and *Gli3*^−/−^ FTOCs.** (A-G) WT (*n*=4) and *Gli3*^−/−^ (*n*=6) E17.5 FTOCs were treated with rHhip or left untreated (control) for 4 days, and analysed by flow cytometry. (B,D,F,G) Scatter plots show relative mean±s.e.m., where each point represents the percentage of SP4 (circles), SP8 (squares) and DP (crosses) populations of one rHhip-treated lobe from an individual thymus, divided by the percentage of that population from the untreated lobe from the same thymus. (A,C) Dot plots of (A) CD8 versus CD4 and (C) CD8 versus CD4 gated on CD3^hi^. (B,D) Percentage of rHhip-treated populations, giving significance by Student's *t*-test compared with untreated control for (B) SP4 (WT, *P*<0.05; *Gli3*^−/−^, *P*<0.03), SP8 (WT, *P*<0.002), DP (WT, *P*<0.006); and for (D) CD3^hi^ SP4 (WT, *P*<0.04; *Gli3*^−/−^, *P*<0.05), CD3^hi^ SP8 (WT, *P*<0.05), CD3^hi^ DP (WT, *P*<0.05). (E) Scatter plots of (left) MFI of CD5 on SP4, DP and SP8 cells and (right) in the same populations gated on CD3^hi^ from a representative experiment. (F) MFI of CD5 in Hhip-treated FTOC on SP4, SP8 and DP showing significance by Student's *t*-test compared with untreated control for SP4 (WT, *P*<0.05); and in (G) MFI of CD5 showing significance by Student's *t*-test compared with untreated control on CD3^hi^ SP4 (WT, *P*<0.002), CD3^hi^ SP8 (WT, *P*<0.0001) and CD3^hi^ DP (*Gli3*^−/−^, *P*<0.05).

Since the Gli3-deficient thymus has increased Hh signalling, loss of Gli3 in the thymus could cause changes that are directly dependent on the increase in the Hh signal or, alternatively, that are dependent on Gli3 but independent of the increase in Hh pathway activation. To investigate whether the differences in the *Gli3*^−/−^ thymus were directly due to increased Hh signalling, we attenuated Hh signalling by treatment with recombinant (r) Hhip to neutralise endogenous Hh proteins in FTOC. As expected, rHhip-treated WT FTOC had a higher proportion of SP4 and SP8 cells but a decreased percentage of DP cells than untreated controls (Fig. 3A,B) (Lau et al., 2017). rHhip-treated *Gli3*^−/−^ FTOC had a significantly higher proportion of SP4 cells (Fig. 3A,B). The mature CD3^hi^ SP4 and CD3^hi^ SP8 populations were significantly increased and the CD3^hi^ DP population decreased in the rHhip-treated WT FTOC relative to their controls, whereas in the rHhip-treated *Gli3*^−/−^ FTOC, although the mean proportional change in both the CD3^hi^ SP4 and CD3^hi^ SP8 populations was greater than in the WT, only the increase in the CD3^hi^ SP4 was significant, and variability was greater (Fig. 3C,D). This increased variability in response to Hh neutralisation in the *Gli3*^−/−^ compared with WT suggested that Gli3 might be required for normal interpretation of changes in the Hh signal.

Neutralisation of Hh proteins also increased cell-surface CD5 expression in WT FTOC (Fig. 3E-G). As expected, the highest cell-surface CD5 expression was observed in the SP4 population in all cultures, and gating on CD3^hi^ cells showed that the CD3^hi^ DP populations expressed lower levels of cell-surface CD5 than the WT CD3^hi^ SP4 and CD3^hi^ SP8 populations (Fig. 3E). Interestingly, rHhip treatment significantly increased the MFI of CD5 on the CD3^hi^ DP population in the *Gli3*^−/−^ FTOC, whereas in WT FTOC MFI of CD3^hi^ SP4 and CD3^hi^ SP8 populations was significantly increased (Fig. 3E-G).

These experiments suggest that the decrease in SP4 differentiation in the *Gli3*^−/−^ FTOC at this developmental transition is a direct result of the increase in Shh, but that Gli3 might also be required to respond to changes in the Shh signal. Consistent with this, previous studies showed that Shh treatment of WT FTOC decreases the SP4 population, the SP4:SP8 ratio and cell-surface CD5 expression, and that in mature T-cells constitutive activation of Gli2-mediated transcription reduces TCR signal transduction (Furmanski et al., 2015, 2012; Rowbotham et al., 2007). By contrast, constitutive loss of Shh, Gli1 or Gli2 from fetal thymus increases differentiation from the DP to SP stage (Drakopoulou et al., 2010; Rowbotham et al., 2007).

### Gli3 expression in TECs plays a key role in T-cell development

We next tested if the changes in thymocyte selection and maturation were the result of the activity of Gli3 expressed in TECs, rather than of cell-intrinsic Gli3 activity in developing thymocytes. We compared fetal thymocyte development in conditional knockouts, in which *Gli3* is conditionally deleted from TECs (*Gli3*^fl/fl^ *FoxN1Cre*^+^), and in which *Gli3* is specifically deleted from all haematopoietic cells including all thymocytes (*Gli3*^fl/fl^ *VavCre*^+^). Interestingly, most of the changes observed in the Gli3-deficient thymus were due to loss of Gli3 expression from TECs.

**Fig. 4.**
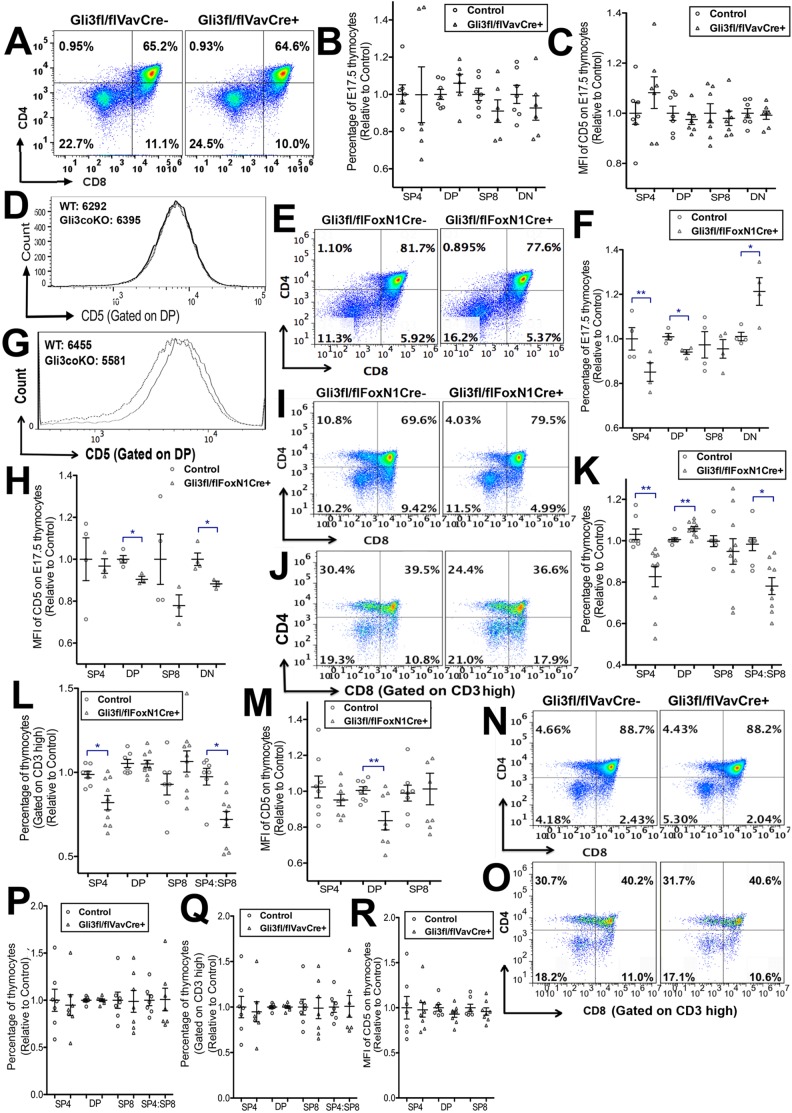
**Gli3 expression in TECs and not thymocyte-intrinsic Gli3 expression is required for SP4 development.** (A-D) Flow cytometry of fresh E17.5 *Gli3*^fl/fl^ *VavCre*^−^ control (*n*=7) and *Gli3*^fl/fl^ *VavCre*^+^ (*n*=6) thymus. Scatter plots show relative mean±s.e.m., where each point represents thymus from a different embryo for control littermate and *Gli3*^fl/fl^ *VavCre*^+^. (A) CD4 and CD8 expression. (B) Relative percentage of thymocyte subsets. (C) Relative MFI of anti-CD5 staining on thymocyte subsets. (D) Representative histogram showing anti-CD5 staining on the DP population in control (solid line) and *Gli3*^fl/fl^ *VavCre*^+^ (dotted line), showing MFIs. No significant differences were found between control and *Cre*^+^ thymi. (E-H) Flow cytometry of fresh E17.5 thymus from *Gli3*^fl/fl^ *FoxN1Cre*^−^ (control, *n*=4) and *Gli3*^fl/fl^ *FoxN1Cre*^+^ (*n*=4) littermates. Scatter plots show relative mean±s.e.m., where each point represents thymus from a different embryo for control and *Gli3*^fl/fl^ *FoxN1Cre*^+^. (E) CD8 against CD4. (F) Relative mean percentage of SP4, DP, SP8 and DN populations, giving significance by Student's *t*-test compared with control littermate for SP4 (*P*<0.01), DP (*P*<0.05) and DN (*P*<0.04). (G) Representative histogram shows CD5 staining on DP thymocytes from control (*Cre*^−^, solid line) and *Cre*^+^ (dotted line). MFI of CD5 fluorescence is given. (H) Relative mean MFI of CD5 on SP4, DP, SP8 and DN populations. Differences are significant between *Cre*^+^ and control for DP (*P*<0.03) and DN (*P*<0.05). (I-M) Flow cytometry analysis of E17.5 FTOC +4 days from *Gli3*^fl/fl^ *FoxN1Cre*^−^ (*n*=8) and *Gli3*^fl/fl^ *FoxN1Cre*^+^ (*n*=10) littermates. Scatter plots show relative mean±s.e.m., where each point represents thymus from a different embryo for control FTOC and *Gli3*^fl/fl^ *FoxN1Cre*^+^ FTOC. (I) CD8 against CD4. (J) CD8 against CD4, gated on CD3^hi^. (K) Relative percentage of SP4, DP and SP8 populations and SP4:SP8 ratio, with significance by Student's *t*-test compared with *Cre*^−^ littermate for SP4 (*P*<0.02), DP (*P*<0.007) and SP4:SP8 ratio (*P*<0.05). (L) Relative percentage of thymocyte populations and CD4:CD8 ratio, gated on CD3^hi^, with significance compared with *Cre*^−^ littermate, for CD3^hi^ SP4 (*P*<0.05) and CD3^hi^ SP4:SP8 ratio (*P*<0.04). (M) Relative MFI of CD5 on thymocyte populations, with significance compared with *Cre*^−^ for DP (*P*<0.007). (N,O) Flow cytometry of E17.5 FTOC +4 days from control *Gli3*^fl/fl^ *VavCre*^−^ (*n*=7) and *Gli3*^fl/fl^ *VavCre*^+^ (*n*=6) littermates. No significant differences were found between *Cre*^−^ and *Cre*^+^. (N) CD4 against CD8. (O) CD4 against CD8, gated on CD3^hi^. (P) Relative percentage of SP4, DP and SP8 populations and SP4:SP8 ratio. (Q) Gated on CD3^hi^, relative percentage of SP4, DP and SP8 populations and SP4:SP8 ratio. (R) MFI of CD5, gated on thymocyte populations.

We first analysed thymocyte development at E17.5, the day on which mature SP4 cells first arise. Conditional deletion of thymocyte-intrinsic *Gli3* in fresh E17.5 *Gli3*^fl/fl^ *VavCre*^+^ thymus did not result in significant changes in the proportion of thymocyte populations or in cell-surface CD5 expression compared with littermate control thymus (Fig. 4A-D). By contrast, conditional deletion of *Gli3* from TECs in *Gli3*^fl/fl^ *FoxN1Cre*^+^ embryos resulted in a significant decrease in the differentiation of SP4 cells (Fig. 4E,F). On E17.5, in addition to a significant reduction in the emerging SP4 population, we observed a significant decrease in the DP population, and a concomitant increase in the DN population, as previously described in the constitutive *Gli3*^−/−^ thymus (Hager-Theodorides et al., 2005) (Fig. 4E,F). Cell-surface CD5 expression was significantly lower on the DP and DN cells in *Cre*^+^ than in the control, indicating lower pre-TCR and/or TCR signal strength (Fig. 4G,H) (Azzam et al., 1998; Rowbotham et al., 2009; Sahni et al., 2015).

We then cultured E17.5 *Gli3*^fl/fl^ *FoxN1Cre*^+^ and *Gli3*^fl/fl^ *FoxN1Cre*^−^ FTOCs for 4 days to investigate the rate of differentiation at the transition from DP to SP4 cells (Fig. 4I-M). Conditional deletion of *Gli3* from TECs resulted in a significant decrease in the proportion of SP4 cells and in the SP4:SP8 ratio, while the proportion of DP cells significantly increased compared with the *Cre*^−^ littermate control (Fig. 4I,K). Gating on CD3^hi^ cells, we observed a significant decrease in the SP4 population and in the SP4:SP8 ratio (Fig. 4J,L). Cell-surface CD5 expression was significantly decreased in the DP population from *Gli3*^fl/fl^ *FoxN1Cre*^+^ FTOC compared with *Cre*^−^ littermates (Fig. 4M). By contrast, FTOC from *Gli3*^fl/fl^ *VavCre*^+^ showed no differences in the rate of differentiation, distribution of thymocyte subsets, or cell-surface CD5 expression compared with control, confirming the importance of Gli3 expression in TECs, rather than in the hematopoietic compartment of the thymus, for the normal regulation of thymocyte differentiation (Fig. 4N-R).

**Fig. 5.**
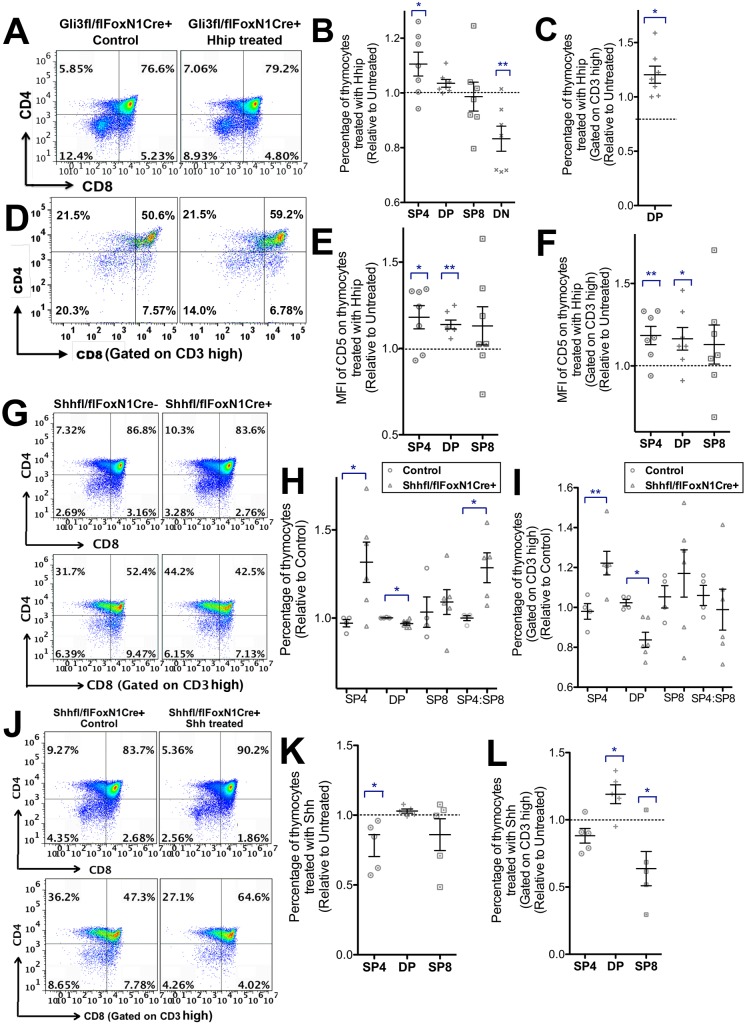
**Neutralisation of Hh in *Gli3*^fl/fl^*FoxN1Cre*^+^ FTOC and conditional deletion of *Shh* from TECs increase SP4 differentiation.** (A-L) Flow cytometry analysis with scatter plots showing relative mean±s.e.m., where each point represents thymus from a different embryo. (A-E) E17.5 *Gli3*^fl/fl^ *FoxN1Cre*^+^ FTOC treated with rHhip for 4 days (*n*=7) compared with the untreated lobe (control) from the same thymus (*n*=7). (A) CD8 against CD4. (B) Percentage of thymocyte populations, showing significance by Student's *t*-test for rHhip treated versus control untreated for SP4 (*P*<0.02) and DN (*P*<0.006). (C) The percentage of DP thymocytes, gated on CD3^hi^. The difference in percentage of CD3^hi^ DP cells between rHhip treated and control was statistically significant by Student's *t*-test (*P*<0.02). (D) CD8 versus CD4, gated on CD3^hi^. (E) MFI of CD5 on rHhip-treated FTOC showing significance by Student's *t*-test versus control untreated FTOC for SP4 (*P*<0.02) and DP (*P*<0.007). (F) Gated on CD3^hi^, MFI of CD5 on rHhip treated showing significance by Student's *t*-test versus control untreated FTOC for CD3^hi^ SP4 (*P*<0.01) and CD3^hi^ DP (*P*<0.02). (G-I) Flow cytometry of E17.5 FTOC +4 days from *Shh*^fl/fl^ *FoxN1Cre*^−^ (*n*=4, control) and *Shh*^fl/fl^ *FoxN1Cre*^+^ (*n*=6) littermates. Scatter plots show relative mean±s.e.m., where each point represents thymus from a different embryo. (H) Percentage of thymocyte populations and SP4:SP8 ratio, in control and *Shh*^fl/fl^ *FoxN1Cre*^+^, giving significance by Student's *t*-test compared with *Cre*^−^ for SP4 (*P*<0.02), DP (*P*<0.04) and SP4:SP8 ratio (*P*<0.05). (I) Percentage of thymocyte populations and SP4:SP8 ratio gated on CD3^hi^, in control and *Shh*^fl/fl^ *FoxN1Cre*^+^, giving significance by Student's *t*-test compared with *Cre*^−^ for CD3^hi^ SP4 (*P*<0.003) and CD3^hi^ DP (*P*<0.002). (J-L) E17.5 *Shh*^fl/fl^ *FoxN1Cre*^+^ FTOC treated with 1 μg/ml rShh for 4 days compared with control untreated *Shh*^fl/fl^ *FoxN1Cre*^+^ FTOC thymus lobe from the same thymus (*n*=5). (J) CD8 against CD4 staining (top); CD8 against CD4 staining, gated on CD3^hi^ (bottom). (K) Percentage of thymocyte population, divided by the percentage from the control untreated cultured lobe from the same thymus, giving significance compared with untreated for SP4 (*P*<0.04). (L) Percentage of thymocyte population, gated on CD3^hi^, divided by the percentage from the control untreated cultured lobe from the same thymus, giving significance compared with untreated control for CD3^hi^ DP (*P*<0.02) and CD3^hi^ SP8 (*P*<0.03).

Since constitutive loss of Gli3 led to Hh-dependent changes in thymocyte differentiation and maturation, we tested whether the changes that resulted from conditional deletion of *Gli3* specifically from TECs were also Hh dependent. We treated the *Gli3*^fl/fl^ *FoxN1Cre*^+^ FTOC with rHhip and observed a significant increase in the SP4 population and a significant decrease in the DN population compared with untreated FTOC (Fig. 5A,B). Gating on CD3^hi^ cells also showed that the rHhip-treated *Gli3*^fl/fl^ *FoxN1Cre*^+^ FTOC had an increased proportion of CD3^hi^ DP cells compared with untreated controls (Fig. 5C,D). The MFI of CD5 on SP4 and DP cells in the rHhip-treated *Gli3*^fl/fl^ *FoxN1Cre*^+^ FTOC was significantly increased compared with the untreated control (Fig. 5E,F). This was in contrast to the effect of rHhip treatment on constitutive *Gli3*^−/−^ FTOC, in which, although rHhip treatment increased the proportion of SP4 cells, it only increased the MFI of anti-CD5 staining in the CD3^hi^ DP population (Fig. 3G). As thymocytes in the *Gli3*^fl/fl^ *FoxN1Cre*^+^ FTOC express Gli3, whereas those in the *Gli3*^−/−^ FTOC do not, this difference suggests that Gli3 activity in developing fetal thymocytes is also required for interpretation of the change in the Shh signal, when the high Shh signal caused by Gli3 deficiency is neutralised by rHhip treatment.

Conditional deletion of *Shh* from TECs (*Shh*^fl/fl^ *FoxN1Cre*^+^) led to the opposite phenotype to conditional deletion of *Gli3* from TECs (Fig. 5G-L). There was a significant increase in the proportion of SP4 cells and in the SP4:SP8 ratio, but a significant decrease in the DP population compared with *Cre*^−^ control FTOC (Fig. 5G,H). The proportion of mature CD3^hi^ SP4 cells was also significantly higher, whereas the proportion of CD3^hi^ DP cells was significantly lower, in *Shh*^fl/fl^ *FoxN1Cre*^+^ FTOC compared with *Cre*^−^ controls (Fig. 5G,I). Treatment of *Shh*^fl/fl^ *FoxN1Cre*^+^ FTOC with rShh reversed this effect and significantly reduced differentiation to SP4 and CD3^hi^ SP8 but increased the proportion of CD3^hi^ DP cells (Fig. 5J-L).

### Transcriptional mechanisms at the DP to SP transition in the Gli3-deficient thymus

**Fig. 6.**
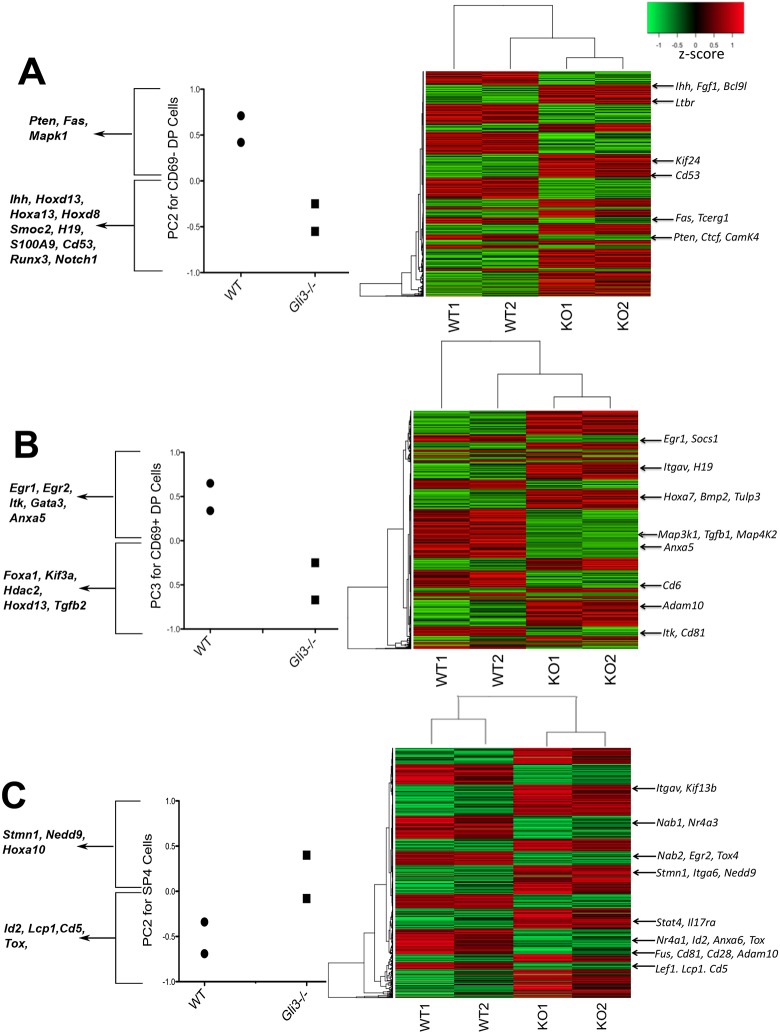
**Microarray datasets show transcriptional differences in Hh signalling genes and thymic selection genes between E18.5 WT and *Gli3* mutant sorted CD69^−^ DP, CD69^+^ DP and SP4 populations.** (A) (Left) PCA axis 2 (PC2) for WT and *Gli3*^−/−^ CD69^−^ DP, showing some genes that contributed to the negative (*Gli3*^−/−^) and positive (WT) PC2 axis. (Right) Heatmap of DEGs identified by intersection analysis between WT and *Gli3*^−/−^ CD69^−^ DP. (B) (Left) PC3 for WT and *Gli3*^−/−^ CD69^+^ DP, showing some genes that contributed to the negative (*Gli3*^−/−^) and positive (WT) axis. (Right) Heatmap of DEGs identified by intersection analysis between WT and *Gli3*^−/−^ CD69^+^ DP. (C) (Left) PC2 for WT and *Gli3*^−/−^ SP4, showing some genes that contributed to the negative (WT) and positive (*Gli3*^−/−^) axis. (Right) Heatmap of DEGs highlighted in intersection analysis between WT and *Gli3*^−/−^ SP4.

In order to understand the transcriptional mechanisms by which Gli3 regulates the DP to SP4 transition and thymic selection, we performed microarrays on FACS-sorted CD69^−^ DP, CD69^+^ DP and SP4 thymocytes from E18.5 WT and *Gli3* mutant thymus (Fig. 6A-C, Fig. S2; GEO accession GSE87499). We identified differentially expressed genes (DEGs) by Ebayes between *Gli3*^−/−^ and WT samples for each sorted population, and used principal component analysis (PCA) to investigate the datasets in an unbiased way (Fig. S2, Tables S1 and S2). For each sorted population dataset, we intersected the 2000 most significant DEGs with the 4000 genes that contributed most to the principal component (PC) axis in which *Gli3*^−/−^ samples segregated from WT, in order to identify genes of interest.

PCA on the CD69^−^ DP microarray data segregated the samples by genotype on PC2, which accounted for 19% of variability (Fig. 6A, Fig. S2A). Interestingly, genes that contributed to PC2, which were higher in the *Gli3*^−/−^ samples than in WT, included many Hh signalling and target genes, consistent with increased Hh signalling in *Gli3*^−/−^. These included *Hoxd13*, *Hoxa13*, *Hoxd8*, *Smoc2*, *H19*, *S100a9* and *Ihh* (Chan et al., 2014; Lu et al., 2015; Mandhan et al., 2006; Outram et al., 2009; Pazin and Albrecht, 2009). *Runx3* and *Notch1*, which both promote SP8 differentiation over SP4, also contributed to PC2, with higher expression in *Gli3*^−/−^ than in WT (Bosselut, 2004; Fowlkes and Robey, 2002). Genes that contributed to PC2, which were more highly expressed in WT samples, included genes important in selection and SP4 maturation, including *Map3k1*, which is required for positive selection (Germain, 2002; Xue et al., 2008) (Fig. 6A).

The intersection analysis of the CD69^−^ DP data highlighted 1083 genes, including the Hh target genes *Fgf1* and *Kif24*, the Wnt pathway activator *Bcl9l* (Martin, 1998; Sarkar et al., 2010), and *Cd53*, which is induced upon a lower affinity TCR-MHC interaction (Puls et al., 2002). These were more highly expressed in *Gli3*^−/−^ than in WT (Fig. 6A, Table S2). In addition, many genes involved in TCR signalling and apoptosis during repertoire selection were identified, including *Fas*, *Tcerg1*, *Ltbr*, *Pten*, *Camk4* and *Ctcf* (Castro et al., 1996; Heath et al., 2008; Krebs et al., 1997; Montes et al., 2015; Zhu et al., 2010), all of which showed lower expression in *Gli3*^−/−^ than in WT.

PCA on the microarray data from the CD69^+^ DP population, which are cells that have received the TCR signal for positive selection (Ge and Chen, 1999), segregated the *Gli3*^−/−^ samples from the WT on PC3, which accounted for 20% of variability (Fig. 6B, Fig. S2B). Again, Hh signalling and target genes, such as *Foxa1*, *Kif3a*, *Hdac2* and *Tgfb2*, contributed to this PC, with higher expression in *Gli3*^−/−^ than in WT (Furmanski et al., 2013; Katoh and Katoh, 2009) (Fig. 6B). By contrast, genes that contributed to PC3 and were more highly expressed in WT than in *Gli3*^−/−^ again included several genes involved in selection and SP4 commitment, including: *Egr2*, which is downstream of TCR signalling and involved in positive selection (Lauritsen et al., 2008); and *Gata3*, an SP4 commitment gene (Hernández-Hoyos et al., 2003).

The intersection analysis of the CD69^+^ DP data identified 1150 genes (Fig. 6B), including some known Hh targets, which were more highly expressed in *Gli3*^−/−^ than WT, such as *Hoxa7*, *Bmp2*, *Tulp3*, *Itgav* and *H19* (Chan et al., 2014). Many genes crucial for thymic selection showed lower expression in *Gli3*^−/−^ than in WT, including: *Itk*, a tyrosine kinase downstream of TCR that is required for positive selection and the SP4:SP8 lineage decision (Lucas et al., 2003); *Egr1*, which is downstream of TCR signalling and involved in positive selection (Lauritsen et al., 2008); *Socs1*, the deficiency of which promotes SP8 differentiation (Ilangumaran et al., 2010); *Anxa5*, which promotes apoptosis during negative selection (Rosenbaum et al., 2011); and *Cd6*, a co-stimulatory molecule that interacts with its ligand on TECs to promote differentiation to SP (Singer et al., 2002).

Finally, we carried out PCA and DEG analysis of the SP4 microarray data. PCA showed that the *Gli3*^−/−^ SP4 data segregated from the WT on PC2, which accounted for 22% of variability (Fig. 6C, Fig. S2). The intersection analysis of the SP4 data highlighted 703 genes. *Egr2* and *Nab2*, which are required for selection, *Anxa6*, which regulates selection-related apoptosis, and *Lef1* and *Tox*, which promote SP4 lineage commitment and maturation, were expressed at lower levels in *Gli3*^−/−^ than in WT (Collins et al., 2006; Rosenbaum et al., 2011)*. Nr4a1*, *Nr4a3* and *Cd5*, which are transcriptional targets of TCR signal transduction with a level of expression that correlates with TCR signal strength, were also lower in *Gli3*^−/−^ than in WT (Moran et al., 2011; Sekiya et al., 2013). Several Hh target genes, such as *Itgav*, *Stmn1*, *Itga6* and *Nedd9*, were more highly expressed in *Gli3*^−/−^ than in WT (Aquino et al., 2009; Lu et al., 2015) (Fig. 6C).

**Fig. 7.**
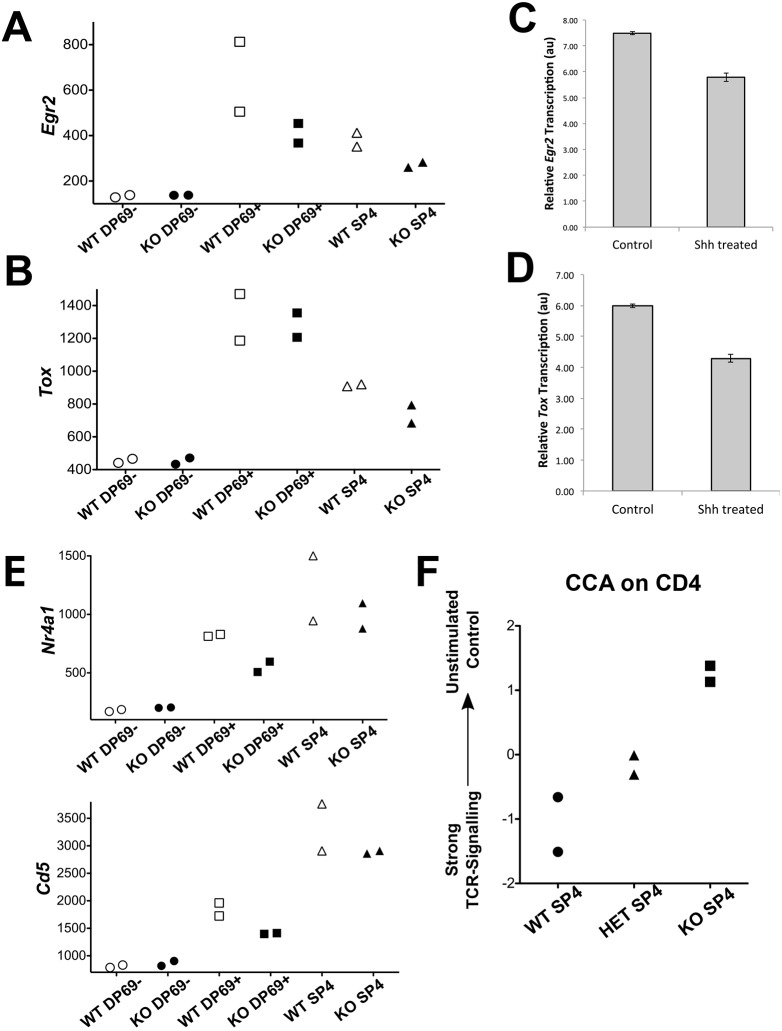
**Microarray and QRT-PCR analyses of transcriptional differences in thymic selection genes in sorted populations from WT and *Gli3* mutant thymus and in rShh-treated WT FTOC.** (A,B) Normalised microarray transcript expression in *Gli3*^−/−^ and WT datasets of (A) *Egr2* and (B) *Tox.* (C,D) QRT-PCR analysis showing a representative experiment (of three) for expression of (C) *Egr2* and (D) *Tox* in thymocytes prepared from E17.5 WT control and rShh-treated FTOCs for 2 days. Differences between control and rShh treated were significant: *Egr2* (*P*<0.02), *Tox* (*P*<0.07). Transcripts were normalised relative to *Hprt.* (E) Normalised microarray transcript expression in *Gli3*^−/−^ and WT datasets of *Nr4a1* (top) and *Cd5* (bottom). (F) CCA showing the separation of the WT, *Gli3*^+/−^ (HET) and *Gli3*^−/−^ (KO) SP4 microarray datasets on a scale of strong to weak TCR stimulation.

Since Hh target genes were upregulated in the *Gli3*^−/−^ CD69^+^ DP population (which is undergoing positive selection), and the FTOC experiments suggested that the reduced DP to SP4 transition in the *Gli3*^−/−^ thymus was due to increased Hh signalling, we tested whether the transcription of genes required for differentiation to SP could be modulated by rShh treatment. We chose *Egr2* and *Tox* for this experiment because both were expressed at very similar low levels in WT and *Gli3*^−/−^ in the CD69^−^ DP dataset, but upregulated in the CD69^+^ DP and SP4 populations. Furthermore, both transcripts were significantly lower in the *Gli3*^−/−^ CD69^+^ DP and *Gli3*^−/−^ SP4 datasets than in their WT counterparts, and these are the populations that contain cells that are undergoing selection (Fig. 7A,B). After 2 days of rShh treatment of WT E17.5 FTOC, the levels of *Egr2* and *Tox* were reduced compared with the untreated control (Fig. 7C,D).

In the microarray data, we observed the same pattern of expression for the known transcriptional target genes of TCR signal transduction, *Nr4a1* and *Cd5* (Fig. 7E). Both were low in CD69^−^ DP cells, upregulated in the CD69^+^ DP population, and again in the SP4 population, but were significantly lower in the *Gli3*^−/−^ CD69^+^ DP and SP4 datasets than in WT. Thus, the differences in expression in these genes only became apparent after TCR signalling for positive selection, in support of the idea that *Gli3* mutation influences SP4 T-cell development by increasing Shh, which signals to DP thymocytes to dampen TCR signal transduction during positive selection.

Taken together, the transcriptome data suggest that cells undergoing selection in the *Gli3*^−/−^ thymus have a lower average strength of TCR signal. To test this, we used canonical correspondence analysis (CCA) to compare the patterns of gene expression in our SP4 datasets with transcriptome data from publically available datasets that were prepared from thymocytes that were receiving different strengths of TCR signals during selection (GSE38909; Lo et al., 2012). We selected the 1500 most significant DEG genes from the GSE38909 dataset and used these to generate a scale of strong to weak TCR signalling and plotted our SP4 datasets against this scale. This analysis showed that the SP4 *Gli3*^−/−^ samples have the transcriptional signature of thymocytes receiving a lower TCR signal than those of WT, whereas the transcriptome of the SP4 population from the *Gli3*^+/−^ thymus showed an intermediate transcriptional signature (Fig. 7F). The CCA therefore confirms that Gli3 is important for selection and differentiation from DP to SP4, and is consistent with higher Shh expression dampening the TCR signal during repertoire selection in the *Gli3*^−/−^ thymus.

## DISCUSSION

Here, we showed that expression of the transcription factor Gli3 in TECs is necessary for normal differentiation from DP to mature SP4 thymocyte in the fetal thymus. Constitutive deletion of *Gli3* reduced differentiation and maturation of SP4 T-cells, and this reduction in differentiation to SP4 was also seen when *Gli3* was conditionally deleted from TECs only, but not when *Gli3* was conditionally deleted from thymocytes. Gli3 repressed expression of Shh, and analysis of an Hh reporter line showed that the Hh signalling pathway was active in developing thymocytes, and that activation of the pathway in thymocytes was increased when *Gli3* was deleted. DP and SP thymocyte populations from *Gli3*^−/−^ had reduced levels of cell-surface CD5, indicative of lower TCR signalling, and consistent with the fact that rShh treatment of WT FTOC and constitutive activation of Hh-mediated transcription both reduced cell-surface CD5 expression (Furmanski et al., 2012; Rowbotham et al., 2007). Conditional deletion of *Shh* from TECs increased differentiation from DP to SP, whereas differentiation from DP to SP4 in the *Gli3* mutant fetal thymus was restored by neutralisation of endogenous Hh proteins. Taken together, our findings indicate that Gli3 activity in TECs promotes SP4 T-cell development by repression of Shh, which signals directly to developing T-cells to reduce TCR signal strength. In support of this, the transcriptome data showed reduced expression of genes important for TCR signalling and positive selection, and of transcriptional targets of TCR signal transduction in the *Gli3*^−/−^ populations compared with their WT counterparts, whereas the WT samples had decreased expression of Hh target and signalling genes. Overall, the *Gli3*^−/−^ SP4 cells had the transcriptional signature of thymocytes that have received a weaker TCR signal than their WT counterparts. The influence of *Gli3* mutation on the SP4 population is consistent with this mechanism, as positive selection to the SP4 lineage requires stronger and longer TCR signals (Bosselut, 2004; Klein et al., 2014; Starr et al., 2003).

Gli3 is expressed in fetal but not adult thymocytes (Hager-Theodorides et al., 2005), and although conditional deletion of *Gli3* from thymocytes did not significantly influence the proportions of DP and SP populations, our experiments showed that Gli3 activity in thymocytes is required for the normal interpretation of changes in the Hh signal. In the constitutive *Gli3*^−/−^ thymus, where increased Shh signalling to developing thymocytes reduced differentiation from DP to SP, although neutralisation of the Hh signal with rHhip treatment was able to restore the proportion of the SP4 population, it only significantly increased cell-surface expression of CD5 in the CD3^hi^ DP population. In contrast to the constitutive *Gli3*^−/−^ thymus, rHhip treatment in *Gli3*^fl/fl^ *FoxN1Cre^+^* FTOC increased cell-surface CD5 expression on SP4 and CD3^hi^ DP populations, suggesting that the ability of developing fetal thymocytes to respond to the decrease in the Shh signal upon rHhip treatment requires thymocyte intrinsic Gli3 activity.

In summary, we showed that expression of the transcription factor Gli3 by TECs is required for normal SP4 T-cell development in the fetal thymus. As Gli3 deficiency increases Shh expression in the thymus, and Hh plays a role in T-cell acute lymphoblastic leukaemia (T-ALL), which arises in the thymus, it will be important to investigate the impact of Gli3 activity in the thymic stromal environment on T-ALL (Dagklis et al., 2016, 2015; González-Gugel et al., 2013; Hou et al., 2014). It will also be important to investigate the influence of Gli3 on shaping the TCR repertoire and in autoimmunity.

## MATERIALS AND METHODS

### Mice

Mice were on a C57BL/6 background. C57BL/6 mice were purchased from Charles River; *Gli3*^fl/fl^, *Gli3*^+/−^ and *Shh*^fl/fl^ from The Jackson Laboratories; GBS-GFP-tg was provided by J. Briscoe (Balaskas et al., 2012); Vav-iCre-tg was provided by D. Kioussis (de Boer et al., 2003); and FoxN1Cre-tg by G. Hollander (Zuklys et al., 2009). As FoxN1-Cre-tg and Vav-Cre-tg can be expressed in the male germline, all conditional knockouts were generated by crossing *Cre*^+^ females with *Cre*^−^ males (Joseph et al., 2013; Shi et al., 2016). Mice were bred and maintained at UCL under UK Home Office regulations.

### Flow cytometry, antibodies and cell purification

Thymus cell suspensions were prepared and stained as described (Hager-Theodorides et al., 2005) using combinations of the following directly conjugated antibodies at concentration of 1:100: (from BD Pharmingen) anti-γδPE (catalogue no. 553178); from eBioscience: anti-TCRβFITC (catalogue no. 11-5961-85), antiCD3PE (catalogue no. 12-0031-82), anti-CD24PE (catalogue no. 12-0241-82) and anti-CD69FITC (catalogue no. 11-0691-85); (from Biolegend) anti-CD3FITC (catalogue no. 100204), anti-CD5FITC (catalogue no. 100605), anti-Qa2FITC (catalogue no. 121709), anti-CD4APC (catalogue no. 116014), anti-CD5PE (catalogue no. 100607), anti-CD8PerCP/Cy5.5 (catalogue no. 100734), anti-CD4PerCP/Cy5.5 (catalogue no. 100539) and anti-CD8APC (catalogue no. 100712). Data were acquired on a C6 Accuri flow cytometer (BD Biosciences) and analysed using FlowJo software. Live cells were gated by FSC and SSC profiles. Data represent at least three experiments.

### Fetal thymus organ cultures

FTOCs were carried out as described (Saldaña et al., 2016). In some experiments, rHhip or rShh (R&D Systems) was added at 1 µg/ml. To allow comparison between litters for statistical analysis, relative numbers or percentages for each genotype or treatment were calculated by dividing by the mean of controls from the same litter (untreated control or WT littermates).

### Microarray and data analysis

E18.5 WT, *Gli3*^+/−^ and *Gli3*^−/−^ thymocytes (*n*=2) were stained for CD4, CD8 and CD69. SP4, CD69^−^ DP and CD69^+^ DP populations were FACS sorted. RNA was extracted using the Arcturus PicoPure RNA Isolation kit (Applied Biosystems) and quantity and quality determined by Bioanalyzer 2100 (Agilent).

Microarrays were performed by UCL Genomics on the Affymetrix GeneChip Mouse Gene 2.0ST Array using standard Ambion (Invitrogen) chemistry. Array data were normalised using the oligo package from R. Data are publically available at GEO (GSE87499).

DEGs were determined using the moderated Ebayes *t*-statistic *P*<0.05 from the limma package in Bioconductor. Principal component analysis (PCA) was performed using normalised microarray transcript expression values, using the CRAN package ade4. PCA is a multivariate statistical method, which can be used to segregate genome-wide transcription datasets according to variability in transcript expression values, taking into account all genes (Ringnér, 2008). PCA can thus cluster microarray datasets to detect dominant patterns of gene expression, as represented by the principal components (PCs).

Canonical correspondence analysis (CCA) is a multivariate analysis that allows the comparison of experimental transcriptome data with publically available datasets from other laboratories (Ono et al., 2014). CCA was performed using the CRAN package vegan. The GSE38909 dataset was used as the environmental variable and our dataset was regressed onto it. It contains thymocytes stimulated with a positively selecting peptide gp250 and the non-selecting control peptide Hb (Lo et al., 2012). We created a strong TCR signalling axis using the DEGs between the control peptide and the positively selecting peptide and regressed our samples onto this axis (Sahni et al., 2015).

### QRT-PCR

RNA extraction and cDNA synthesis were performed as described (Hager-Theodorides et al., 2005). QRT-PCR, using QuantiTect primers for *Egr2* and *Tox* (Qiagen) and iQSYBR Green Supermix (Bio-Rad), was run on an iCycler (Bio-Rad). Transcripts were normalised relative to *Hprt*.

### ELISA

Shh ELISA was performed using the Shh N-Terminus Quantikine ELISA Kit (R&D Systems). Entire E17.5 thymi were crushed and centrifuged at 3 ***g*** for 5 min, and ELISA was performed on the supernatants.

### PCR for genotyping

DNA extraction and PCR were carried out using methods and primers described previously (Hager-Theodorides et al., 2005; Saldaña et al., 2016).

### Statistical analysis

Statistical analysis was performed using unpaired two-tailed Student's *t*-tests and *P*≤0.05 was considered significant. In figures: *P≤0.05, **P≤0.01 and ***P≤0.001.

## Supplementary Material

Supplementary information

Supplementary information

## References

[DEV146910C1] Aquino J. B., Lallemend F., Marmigère F., Adameyko I. I., Golemis E. A. and Ernfors P. (2009). The retinoic acid inducible Cas-family signaling protein Nedd9 regulates neural crest cell migration by modulating adhesion and actin dynamics. *Neuroscience* 162, 1106-1119. 10.1016/j.neuroscience.2009.05.035 19464348 PMC2797478

[DEV146910C2] Azzam H. S., Grinberg A., Lui K., Shen H., Shores E. W. and Love P. E. (1998). CD5 expression is developmentally regulated by T cell receptor (TCR) signals and TCR avidity. *J. Exp. Med.* 188, 2301-2311. 10.1084/jem.188.12.2301 9858516 PMC2212429

[DEV146910C3] Azzam H. S., DeJarnette J. B., Huang K., Emmons R., Park C.-S., Sommers C. L., El-Khoury D., Shores E. W. and Love P. E. (2001). Fine tuning of TCR signaling by CD5. *J. Immunol.* 166, 5464-5472. 10.4049/jimmunol.166.9.5464 11313384

[DEV146910C4] Balaskas N., Ribeiro A., Panovska J., Dessaud E., Sasai N., Page K. M., Briscoe J. and Ribes V. (2012). Gene regulatory logic for reading the Sonic Hedgehog signaling gradient in the vertebrate neural tube. *Cell* 148, 273-284. 10.1016/j.cell.2011.10.047 22265416 PMC3267043

[DEV146910C5] Barbarulo A., Lau C.-I., Mengrelis K., Ross S., Solanki A., Saldaña J. I. and Crompton T. (2016). Hedgehog signalling in the embryonic mouse thymus. *J. Dev. Biol.* 4, 22 10.3390/jdb4030022 27504268 PMC4975939

[DEV146910C6] Bosselut R. (2004). CD4/CD8-lineage differentiation in the thymus: from nuclear effectors to membrane signals. *Nat. Rev. Immunol.* 4, 529-540. 10.1038/nri1392 15229472

[DEV146910C7] Brugnera E., Bhandoola A., Cibotti R., Yu Q., Guinter T. I., Yamashita Y., Sharrow S. O. and Singer A. (2000). Coreceptor reversal in the thymus: signaled CD4+8+ thymocytes initially terminate CD8 transcription even when differentiating into CD8+ T cells. *Immunity* 13, 59-71. 10.1016/S1074-7613(00)00008-X 10933395

[DEV146910C8] Carpenter A. C. and Bosselut R. (2010). Decision checkpoints in the thymus. *Nat. Immunol.* 11, 666-673. 10.1038/ni.1887 20644572 PMC3388799

[DEV146910C9] Castro J. E., Listman J. A., Jacobson B. A., Wang Y., Lopez P. A., Ju S., Finn P. W. and Perkins D. L. (1996). Fas modulation of apoptosis during negative selection of thymocytes. *Immunity* 5, 617-627. 10.1016/S1074-7613(00)80275-7 8986720

[DEV146910C10] Chan L. H., Wang W., Yeung W., Deng Y., Yuan P. and Mak K. K. (2014). Hedgehog signaling induces osteosarcoma development through Yap1 and H19 overexpression. *Oncogene* 33, 4857-4866. 10.1038/onc.2013.433 24141783

[DEV146910C11] Collins S., Wolfraim L. A., Drake C. G., Horton M. R. and Powell J. D. (2006). Cutting Edge: TCR-induced NAB2 enhances T cell function by coactivating IL-2 transcription. *J. Immunol.* 177, 8301-8305. 10.4049/jimmunol.177.12.8301 17142725

[DEV146910C12] Crompton T., Outram S. V. and Hager-Theodorides A. L. (2007). Sonic hedgehog signalling in T-cell development and activation. *Nat. Rev. Immunol.* 7, 726-735. 10.1038/nri2151 17690714

[DEV146910C13] Dagklis A., Pauwels D., Lahortiga I., Geerdens E., Bittoun E., Cauwelier B., Tousseyn T., Uyttebroeck A., Maertens J., Verhoef G. et al. (2015). Hedgehog pathway mutations in T-cell acute lymphoblastic leukemia. *Haematologica* 100, e102-e105. 10.3324/haematol.2014.119248 25527561 PMC4349289

[DEV146910C14] Dagklis A., Demeyer S., De Bie J., Radaelli E., Pauwels D., Degryse S., Gielen O., Vicente C., Vandepoel R., Geerdens E. et al. (2016). Hedgehog pathway activation in T-cell acute lymphoblastic leukemia predicts response to SMO and GLI1 inhibitors. *Blood* 128, 2642-2654. 10.1182/blood-2016-03-703454 27694322

[DEV146910C15] de Boer J., Williams A., Skavdis G., Harker N., Coles M., Tolaini M., Norton T., Williams K., Roderick K., Potocnik A. J. et al. (2003). Transgenic mice with hematopoietic and lymphoid specific expression of Cre. *Eur. J. Immunol.* 33, 314-325. 10.1002/immu.200310005 12548562

[DEV146910C16] Drakopoulou E., Outram S. V., Rowbotham N. J., Ross S. E., Furmanski A. L., Saldana J. I., Hager-Theodorides A. L. and Crompton T. (2010). Non-redundant role for the transcription factor Gli1 at multiple stages of thymocyte development. *Cell Cycle* 9, 4144-4152. 10.4161/cc.9.20.13453 20935514 PMC3055198

[DEV146910C17] El Andaloussi A., Graves S., Meng F., Mandal M., Mashayekhi M. and Aifantis I. (2006). Hedgehog signaling controls thymocyte progenitor homeostasis and differentiation in the thymus. *Nat. Immunol.* 7, 418-426. 10.1038/ni1313 16518394

[DEV146910C18] Fowlkes B. J. and Robey E. A. (2002). A reassessment of the effect of activated Notch1 on CD4 and CD8 T cell development. *J. Immunol.* 169, 1817-1821. 10.4049/jimmunol.169.4.1817 12165504

[DEV146910C19] Furmanski A. L., Saldana J. I., Rowbotham N. J., Ross S. E. and Crompton T. (2012). Role of Hedgehog signalling at the transition from double-positive to single-positive thymocyte. *Eur. J. Immunol.* 42, 489-499. 10.1002/eji.201141758 22101858 PMC3378705

[DEV146910C20] Furmanski A. L., Saldana J. I., Ono M., Sahni H., Paschalidis N., D'Acquisto F. and Crompton T. (2013). Tissue-derived hedgehog proteins modulate Th differentiation and disease. *J. Immunol.* 190, 2641-2649. 10.4049/jimmunol.1202541 23408837 PMC3672981

[DEV146910C21] Furmanski A. L., Barbarulo A., Solanki A., Lau C.-I., Sahni H., Saldana J. I., D'Acquisto F. and Crompton T. (2015). The transcriptional activator Gli2 modulates T-cell receptor signalling through attenuation of AP-1 and NFkappaB activity. *J. Cell Sci.* 128, 2085-2095. 10.1242/jcs.165803 25908851 PMC4450292

[DEV146910C22] Ge Q. and Chen W. F. (1999). Phenotypic identification of the subgroups of murine T-cell receptor alphabeta+ CD4+ CD8− thymocytes and its implication in the late stage of thymocyte development. *Immunology* 97, 665-671. 10.1046/j.1365-2567.1999.00816.x 10457221 PMC2326876

[DEV146910C23] Germain R. N. (2002). T-cell development and the CD4-CD8 lineage decision. *Nat. Rev. Immunol.* 2, 309-322. 10.1038/nri798 12033737

[DEV146910C24] González-Gugel E., Villa-Morales M., Santos J., Bueno M. J., Malumbres M., Rodríguez-Pinilla S. M., Piris M. A. and Fernández-Piqueras J. (2013). Down-regulation of specific miRNAs enhances the expression of the gene Smoothened and contributes to T-cell lymphoblastic lymphoma development. *Carcinogenesis* 34, 902-908. 10.1093/carcin/bgs404 23288923

[DEV146910C25] Hager-Theodorides A. L., Dessens J. T., Outram S. V. and Crompton T. (2005). The transcription factor Gli3 regulates differentiation of fetal CD4− CD8− double-negative thymocytes. *Blood* 106, 1296-1304. 10.1182/blood-2005-03-0998 15855276 PMC1274277

[DEV146910C26] Hager-Theodorides A. L., Furmanski A. L., Ross S. E., Outram S. V., Rowbotham N. J. and Crompton T. (2009). The Gli3 transcription factor expressed in the thymus stroma controls thymocyte negative selection via Hedgehog-dependent and -independent mechanisms. *J. Immunol.* 183, 3023-3032. 10.4049/jimmunol.0900152 19667090

[DEV146910C27] Heath H., Ribeiro de Almeida C., Sleutels F., Dingjan G., van de Nobelen S., Jonkers I., Ling K.-W., Gribnau J., Renkawitz R., Grosveld F. et al. (2008). CTCF regulates cell cycle progression of alphabeta T cells in the thymus. *EMBO J.* 27, 2839-2850. 10.1038/emboj.2008.214 18923423 PMC2580790

[DEV146910C28] Hernández-Hoyos G., Anderson M. K., Wang C., Rothenberg E. V. and Alberola-Ila J. (2003). GATA-3 expression is controlled by TCR signals and regulates CD4/CD8 differentiation. *Immunity* 19, 83-94. 10.1016/S1074-7613(03)00176-6 12871641

[DEV146910C29] Hou X., Chen X., Zhang P., Fan Y., Ma A., Pang T., Song Z., Jin Y., Hao W., Liu F. et al. (2014). Inhibition of hedgehog signaling by GANT58 induces apoptosis and shows synergistic antitumor activity with AKT inhibitor in acute T cell leukemia cells. *Biochimie* 101, 50-59. 10.1016/j.biochi.2013.12.019 24394624

[DEV146910C30] Ilangumaran S., Gagnon J., Leblanc C., Poussier P. and Ramanathan S. (2010). Increased generation of CD8 single positive cells in SOCS1-deficient thymus does not proportionately increase their export. *Immunol. Lett.* 132, 12-17. 10.1016/j.imlet.2010.04.009 20438760

[DEV146910C31] Joseph C., Quach J. M., Walkley C. R., Lane S. W., Lo Celso C. and Purton L. E. (2013). Deciphering hematopoietic stem cells in their niches: a critical appraisal of genetic models, lineage tracing, and imaging strategies. *Cell Stem Cell* 13, 520-533. 10.1016/j.stem.2013.10.010 24209759

[DEV146910C32] Katoh Y. and Katoh M. (2009). Hedgehog target genes: mechanisms of carcinogenesis induced by aberrant hedgehog signaling activation. *Curr. Mol. Med.* 9, 873-886. 10.2174/156652409789105570 19860666

[DEV146910C33] Klein L., Kyewski B., Allen P. M. and Hogquist K. A. (2014). Positive and negative selection of the T cell repertoire: what thymocytes see (and don't see). *Nat. Rev. Immunol.* 14, 377-391. 10.1038/nri3667 24830344 PMC4757912

[DEV146910C34] Krebs J., Wilson A. and Kisielow P. (1997). Calmodulin-dependent protein kinase IV during T-cell development. *Biochem. Biophys. Res. Commun.* 241, 383-389. 10.1006/bbrc.1997.7823 9425280

[DEV146910C35] Laky K. and Fowlkes B. J. (2008). Notch signaling in CD4 and CD8 T cell development. *Curr. Opin. Immunol.* 20, 197-202. 10.1016/j.coi.2008.03.004 18434124 PMC2475578

[DEV146910C36] Lau C.-I., Barbarulo A., Solanki A., Saldaña J. I. and Crompton T. (2017). The kinesin motor protein Kif7 is required for T-cell development and normal MHC expression on thymic epithelial cells (TEC) in the thymus. *Oncotarget* 8, 24163-24176. 10.18632/oncotarget.15241 28445929 PMC5421836

[DEV146910C37] Lauritsen J.-P. H., Kurella S., Lee S.-Y., Lefebvre J. M., Rhodes M., Alberola-Ila J. and Wiest D. L. (2008). Egr2 is required for Bcl-2 induction during positive selection. *J. Immunol.* 181, 7778-7785. 10.4049/jimmunol.181.11.7778 19017967 PMC2587029

[DEV146910C38] Lo W.-L., Donermeyer D. L. and Allen P. M. (2012). A voltage-gated sodium channel is essential for the positive selection of CD4(+) T cells. *Nat. Immunol.* 13, 880-887. 10.1038/ni.2379 22842345 PMC3426661

[DEV146910C39] Lu Y., Li J., Cheng J. and Lubahn D. B. (2015). Genes targeted by the Hedgehog-signaling pathway can be regulated by Estrogen related receptor beta. *BMC Mol. Biol.* 16, 19 10.1186/s12867-015-0047-3 26597826 PMC4657266

[DEV146910C40] Lucas J. A., Miller A. T., Atherly L. O. and Berg L. J. (2003). The role of Tec family kinases in T cell development and function. *Immunol. Rev.* 191, 119-138. 10.1034/j.1600-065X.2003.00029.x 12614356

[DEV146910C41] Mandhan P., Quan Q. B., Beasley S. and Sullivan M. (2006). Sonic hedgehog, BMP4, and Hox genes in the development of anorectal malformations in Ethylenethiourea-exposed fetal rats. *J. Pediatr. Surg.* 41, 2041-2045. 10.1016/j.jpedsurg.2006.08.035 17161201

[DEV146910C42] Martin G. R. (1998). The roles of FGFs in the early development of vertebrate limbs. *Genes Dev.* 12, 1571-1586. 10.1101/gad.12.11.1571 9620845

[DEV146910C43] Montes M., Coiras M., Becerra S., Moreno-Castro C., Mateos E., Majuelos J., Oliver F. J., Hernandez-Munain C., Alcami J. and Sune C. (2015). Functional consequences for apoptosis by Transcription Elongation Regulator 1 (TCERG1)-mediated Bcl-x and Fas/CD95 alternative splicing. *PLoS ONE* 10, e0139812 10.1371/journal.pone.0139812 26462236 PMC4604205

[DEV146910C44] Moran A. E., Holzapfel K. L., Xing Y., Cunningham N. R., Maltzman J. S., Punt J. and Hogquist K. A. (2011). T cell receptor signal strength in Treg and iNKT cell development demonstrated by a novel fluorescent reporter mouse. *J. Exp. Med.* 208, 1279-1289. 10.1084/jem.20110308 21606508 PMC3173240

[DEV146910C45] Naito T., Tanaka H., Naoe Y. and Taniuchi I. (2011). Transcriptional control of T-cell development. *Int. Immunol.* 23, 661-668. 10.1093/intimm/dxr078 21948191

[DEV146910C46] Ono M., Tanaka R. J. and Kano M. (2014). Visualisation of the T cell differentiation programme by canonical correspondence analysis of transcriptomes. *BMC Genomics* 15, 1028 10.1186/1471-2164-15-1028 25428805 PMC4258272

[DEV146910C47] Outram S. V., Varas A., Pepicelli C. V. and Crompton T. (2000). Hedgehog signaling regulates differentiation from double-negative to double-positive thymocyte. *Immunity* 13, 187-197. 10.1016/S1074-7613(00)00019-4 10981962

[DEV146910C48] Outram S. V., Hager-Theodorides A. L., Shah D. K., Rowbotham N. J., Drakopoulou E., Ross S. E., Lanske B., Dessens J. T. and Crompton T. (2009). Indian hedgehog (Ihh) both promotes and restricts thymocyte differentiation. *Blood* 113, 2217-2228. 10.1182/blood-2008-03-144840 19109233

[DEV146910C49] Park J.-H., Adoro S., Guinter T., Erman B., Alag A. S., Catalfamo M., Kimura M. Y., Cui Y., Lucas P. J., Gress R. E. et al. (2010). Signaling by intrathymic cytokines, not T cell antigen receptors, specifies CD8 lineage choice and promotes the differentiation of cytotoxic-lineage T cells. *Nat. Immunol.* 11, 257-264. 10.1038/ni.1840 20118929 PMC3555225

[DEV146910C50] Pazin D. E. and Albrecht K. H. (2009). Developmental expression of Smoc1 and Smoc2 suggests potential roles in fetal gonad and reproductive tract differentiation. *Dev. Dyn.* 238, 2877-2890. 10.1002/dvdy.22124 19842175 PMC3070464

[DEV146910C51] Puls K. L., Hogquist K. A., Reilly N. and Wright M. D. (2002). CD53, a thymocyte selection marker whose induction requires a lower affinity TCR-MHC interaction than CD69, but is up-regulated with slower kinetics. *Int. Immunol.* 14, 249-258. 10.1093/intimm/14.3.249 11867561

[DEV146910C52] Ramsbottom S. A. and Pownall M. E. (2016). Regulation of Hedgehog signalling inside and outside the cell. *J. Dev. Biol.* 4, 23 10.3390/jdb4030023 27547735 PMC4990124

[DEV146910C53] Ringnér M. (2008). What is principal component analysis? *Nat. Biotechnol.* 26, 303-304. 10.1038/nbt0308-303 18327243

[DEV146910C54] Rosenbaum S., Kreft S., Etich J., Frie C., Stermann J., Grskovic I., Frey B., Mielenz D., Pöschl E., Gaipl U. et al. (2011). Identification of novel binding partners (annexins) for the cell death signal phosphatidylserine and definition of their recognition motif. *J. Biol. Chem.* 286, 5708-5716. 10.1074/jbc.M110.193086 21131363 PMC3037683

[DEV146910C55] Rowbotham N. J., Hager-Theodorides A. L., Cebecauer M., Shah D. K., Drakopoulou E., Dyson J., Outram S. V. and Crompton T. (2007). Activation of the Hedgehog signaling pathway in T-lineage cells inhibits TCR repertoire selection in the thymus and peripheral T-cell activation. *Blood* 109, 3757-3766. 10.1182/blood-2006-07-037655 17227833 PMC1874579

[DEV146910C56] Rowbotham N. J., Hager-Theodorides A. L., Furmanski A. L., Ross S. E., Outram S. V., Dessens J. T. and Crompton T. (2009). Sonic hedgehog negatively regulates pre-TCR-induced differentiation by a Gli2-dependent mechanism. *Blood* 113, 5144-5156. 10.1182/blood-2008-10-185751 19273836 PMC2686185

[DEV146910C57] Sacedón R., Varas A., Hernández-López C., Gutiérrez-deFrías C., Crompton T., Zapata A. G. and Vicente A. (2003). Expression of hedgehog proteins in the human thymus. *J. Histochem. Cytochem.* 51, 1557-1566. 10.1177/002215540305101115 14566027 PMC1249508

[DEV146910C58] Sahni H., Ross S., Barbarulo A., Solanki A., Lau C.-I., Furmanski A., Saldaña J. I., Ono M., Hubank M., Barenco M. et al. (2015). A genome wide transcriptional model of the complex response to pre-TCR signalling during thymocyte differentiation. *Oncotarget* 6, 28646-28660. 10.18632/oncotarget.5796 26415229 PMC4745683

[DEV146910C59] Saldaña J. I., Solanki A., Lau C.-I., Sahni H., Ross S., Furmanski A. L., Ono M., Holländer G. and Crompton T. (2016). Sonic Hedgehog regulates thymic epithelial cell differentiation. *J. Autoimmun.* 68, 86-97. 10.1016/j.jaut.2015.12.004 26778835 PMC4803023

[DEV146910C60] Sarkar F. H., Li Y., Wang Z. and Kong D. (2010). The role of nutraceuticals in the regulation of Wnt and Hedgehog signaling in cancer. *Cancer Metastasis Rev.* 29, 383-394. 10.1007/s10555-010-9233-4 20711635 PMC2974632

[DEV146910C61] Sasaki H., Nishizaki Y., Hui C., Nakafuku M. and Kondoh H. (1999). Regulation of Gli2 and Gli3 activities by an amino-terminal repression domain: implication of Gli2 and Gli3 as primary mediators of Shh signaling. *Development* 126, 3915-3924. 10433919 10.1242/dev.126.17.3915

[DEV146910C62] Sekiya T., Kashiwagi I., Yoshida R., Fukaya T., Morita R., Kimura A., Ichinose H., Metzger D., Chambon P. and Yoshimura A. (2013). Nr4a receptors are essential for thymic regulatory T cell development and immune homeostasis. *Nat. Immunol.* 14, 230-237. 10.1038/ni.2520 23334790

[DEV146910C63] Shah D. K., Hager-Theodorides A. L., Outram S. V., Ross S. E., Varas A. and Crompton T. (2004). Reduced thymocyte development in sonic hedgehog knockout embryos. *J. Immunol.* 172, 2296-2306. 10.4049/jimmunol.172.4.2296 14764698

[DEV146910C64] Shi J., Getun I., Torres B. and Petrie H. T. (2016). Foxn1[Cre] expression in the male germline. *PLoS ONE* 11, e0166967 10.1371/journal.pone.0166967 27880796 PMC5120802

[DEV146910C65] Singer N. G., Fox D. A., Haqqi T. M., Beretta L., Endres J. S., Prohaska S., Parnes J. R., Bromberg J. and Sramkoski R. M. (2002). CD6: expression during development, apoptosis and selection of human and mouse thymocytes. *Int. Immunol.* 14, 585-597. 10.1093/intimm/dxf025 12039910

[DEV146910C66] Singer A., Adoro S. and Park J.-H. (2008). Lineage fate and intense debate: myths, models and mechanisms of CD4- versus CD8-lineage choice. *Nat. Rev. Immunol.* 8, 788-801. 10.1038/nri2416 18802443 PMC2760737

[DEV146910C67] Solanki A., Lau C.-I., Saldaña J. I., Ross S. and Crompton T. (2017). The transcription factor Gli3 promotes B cell development in fetal liver through repression of Shh. *J. Exp. Med.* 214, 2041-2058. 10.1084/jem.20160852 28533268 PMC5502423

[DEV146910C68] Starr T. K., Jameson S. C. and Hogquist K. A. (2003). Positive and negative selection of T cells. *Annu. Rev. Immunol.* 21, 139-176. 10.1146/annurev.immunol.21.120601.141107 12414722

[DEV146910C69] Takahama Y. (2006). Journey through the thymus: stromal guides for T-cell development and selection. *Nat. Rev. Immunol.* 6, 127-135. 10.1038/nri1781 16491137

[DEV146910C70] te Welscher P., Fernandez-Teran M., Ros M. A. and Zeller R. (2002). Mutual genetic antagonism involving GLI3 and dHAND prepatterns the vertebrate limb bud mesenchyme prior to SHH signaling. *Genes Dev.* 16, 421-426. 10.1101/gad.219202 11850405 PMC155343

[DEV146910C71] Vantourout P. and Hayday A. (2013). Six-of-the-best: unique contributions of gammadelta T cells to immunology. *Nat. Rev. Immunol.* 13, 88-100. 10.1038/nri3384 23348415 PMC3951794

[DEV146910C72] Wang B., Fallon J. F. and Beachy P. A. (2000). Hedgehog-regulated processing of Gli3 produces an anterior/posterior repressor gradient in the developing vertebrate limb. *Cell* 100, 423-434. 10.1016/S0092-8674(00)80678-9 10693759

[DEV146910C73] Weinreich M. A. and Hogquist K. A. (2008). Thymic emigration: when and how T cells leave home. *J. Immunol.* 181, 2265-2270. 10.4049/jimmunol.181.4.2265 18684914 PMC2861282

[DEV146910C74] Xue L., Chiang L., Kang C. and Winoto A. (2008). The role of the PI3K-AKT kinase pathway in T-cell development beyond the beta checkpoint. *Eur. J. Immunol.* 38, 3200-3207. 10.1002/eji.200838614 18991293 PMC2614442

[DEV146910C75] Zhu M., Brown N. K. and Fu Y.-X. (2010). Direct and indirect roles of the LTbetaR pathway in central tolerance induction. *Trends Immunol.* 31, 325-331. 10.1016/j.it.2010.06.005 20675191 PMC2933296

[DEV146910C76] Zuklys S., Gill J., Keller M. P., Hauri-Hohl M., Zhanybekova S., Balciunaite G., Na K.-J., Jeker L. T., Hafen K., Tsukamoto N. et al. (2009). Stabilized beta-catenin in thymic epithelial cells blocks thymus development and function. *J. Immunol.* 182, 2997-3007. 10.4049/jimmunol.0713723 19234195

