# Supplementary material for: Gli3 in fetal thymic epithelial cells promotes thymocyte positive selection and differentiation by repression of Shh
Source: Development. 2018 Feb 1;145(3):dev146910. doi: 10.1242/dev.146910 (PMC5817998; doi:10.1242/dev.146910)
Supplement: Supplementary information [file develop-145-146910-s1.pdf]

S1

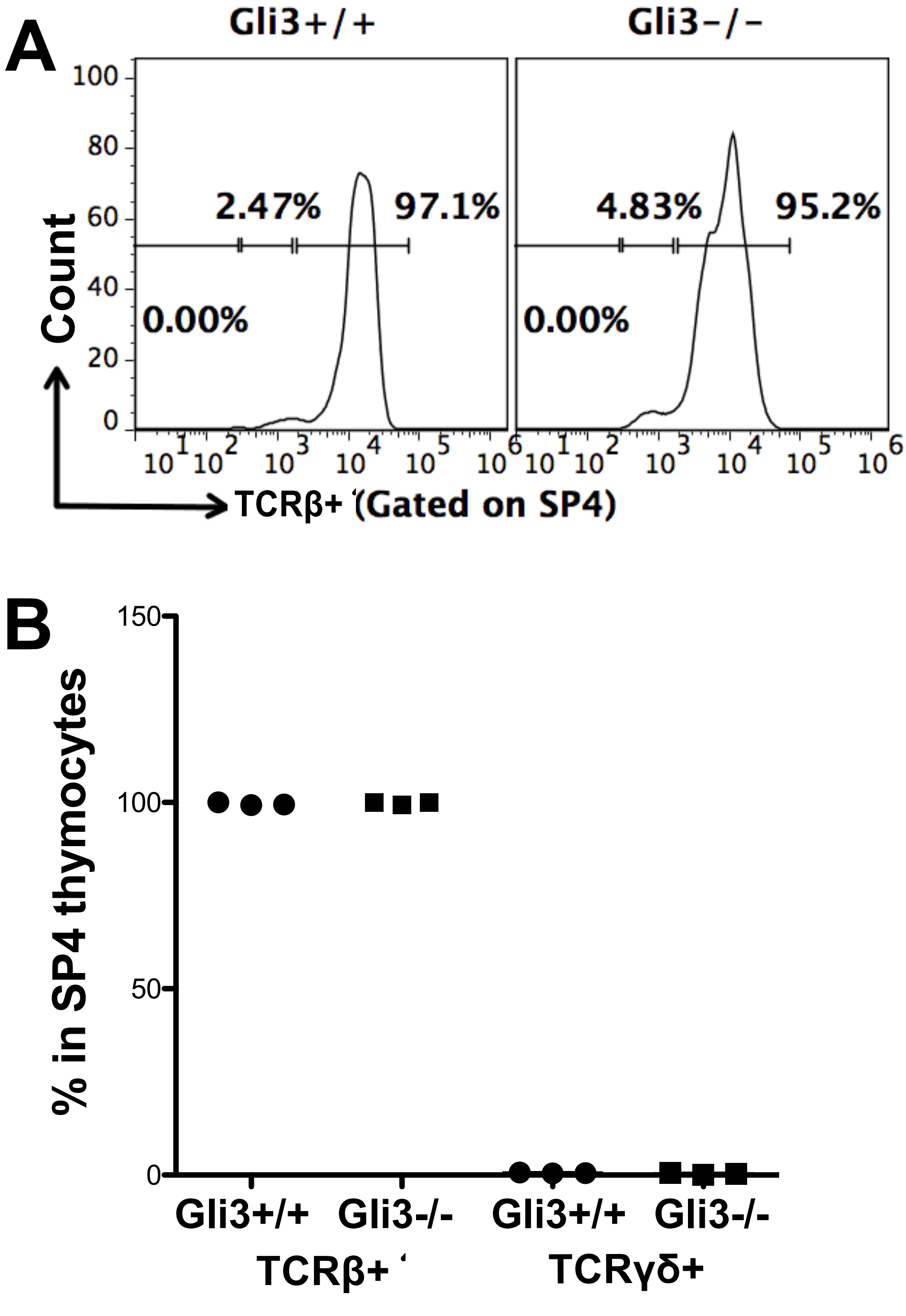

Development • Supplementary information

Figure S1: Expression of TCRβ and TCRγδ in WT and Gli3-deficient E17.5+4 Days FTOCs.

(A) Histograms showing the expression of TCRβ, gated on SP4 cells from Gli3+/+ and Gli3-/-. (B) Scatter plot showing the percentage of TCRβ+ and TCRγδ+ cells in the SP4 population from Gli3+/+ and Gli3-/- (n=3).

Development • Supplementary information

S2

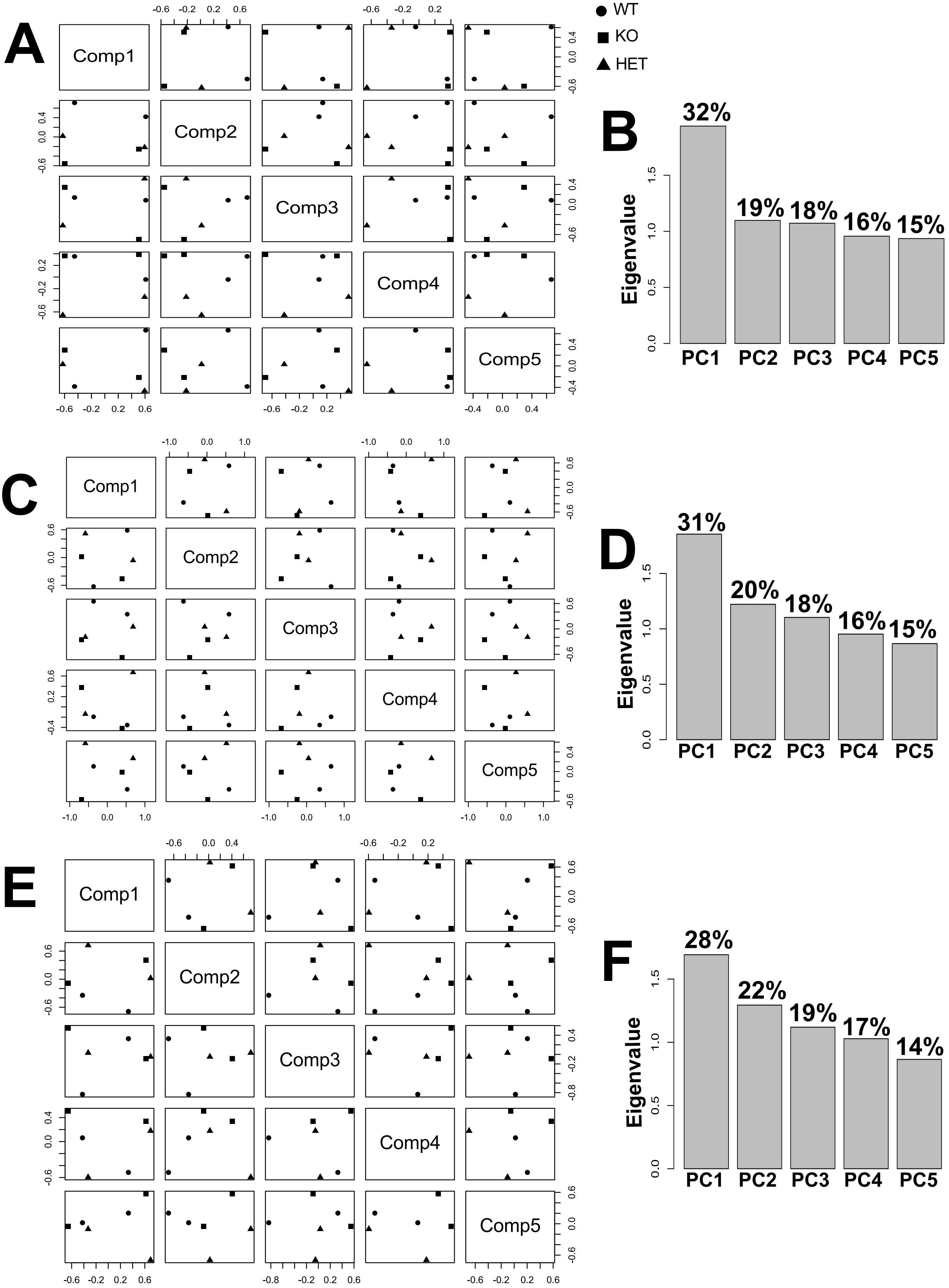

Development • Supplementary information

Figure S2: Principal Component Analyses of E18.5 WT and Gli3-mutant facs-sorted CD69-DP, CD69+DP and SP4 populations.

PCA of the CD69-DP (A), the CD69+DP (C) and SP4 (E) datasets. Eigenvalues and percentage variability associated with each principal component for CD69-DP (B), the CD69+DP (D) and SP4 (F).

Development • Supplementary information

Table S1: Summary of intersected DEG and PCA genes annotated in the heatmap (Figure 6) of known relevant function.

Gene Name	Function in Thymus	Reference
**TCR repertoire selection Genes**		
Egr1	Egr1 is rapidly upregulated after TCR stimulation and increased Egr1 expression increases thymic selection.	(Shao et al., 1997)
Egr2	Egr2-deficiency impairs positive selection of both CD4 and CD8 SP thymocytes. It also upregulates survival molecule Bcl-2 during positive selection allowing survival of positively selected thymocytes	(Lauritsen et al., 2008)
Nab1	The Nab family members interact with the Egr1/2 to regulate different function in thymocytes and T cells.	(Collins et al., 2008; Decker et al., 2003)
Nab2		
Itk	Itk is very importance for the efficient positive and negative selection of thymocytes.	(Andreotti et al., 2010)
Tox	TOX is important for the positive selection towards the CD4+ T cell lineage in the thymus	(Aliahmad et al., 2011)
Lef1	LEF-1 is important for the positive selection of CD4 SP thymocytes from the DP stage. Lef1 directly induce the master regulator of CD4 lineage commitment, ThPok.	(Steinke et al., 2014)
Pten	Loss of Pten leads to defects in negative selection	(Suzuki et al., 2001)
Socs1	Socs1 deficiency leads to impaired positive and negative selection in the thymus. Socs1 is important for CD4 T cell development.	(Catlett and Hedrick, 2005)
Id2	Id2 and Id3 allow CD8+ T cell development	(Jones-Mason et al., 2012)
Fas	Fas-FasL interaction mediates activation-induced cell death of mature postthymic T cells, allowing effective negative selection and elimination of autoimmune cells	(Kishimoto and Sprent, 1997)
CamK4	CaMK4 regulates the Ca2+-dependent gene transcription, and loss of CaMK4 in thymocytes impairs positive selection.	(Raman et al., 2001)
Cd6	Increase in CD6 expression allow DP thymocytes differentiate to a SPs.	(Singer et al., 2002)

Development • Supplementary information

Cd53	CD53 expression is correlated with positive selection. DP thymocytes expressing CD53 are undergoing selection or have just undergone selection.	(Puls et al., 2002)
**TCR signal strength modulators**		
Cd81	Loss of CD81 in thymocytes increases the TCR signal strength	(Cevik et al., 2012)
Cd5	High cell surface CD5 expression correlates with a stronger TCR signal and vice versa	(Azzam et al., 2001)
Nr4a1	The expression of the Nr4a family member is also correlated with increases in TCR signal strength.	(Moran et al., 2011; Nowyhed et al., 2015)
Nr4a3		
**Hedgehog Signalling and Target Genes**		
Ihh	Hedgehog protein expressed in DP cells	(Outram et al., 2009)
Bmp2	Hedgehog signalling target involved in T-cell development in thymus	(Hager-Theodorides et al., 2002; Outram et al., 2000)
Hoxa7	Homeobox (Hox) family members are targets of Hedgehog signalling and interact with various Hh family members to control developmental processes.	(Roberts et al., 1995)
H19	Hh signaling increases H19 expression	(Chan et al., 2014)
Kif13b	Kif13b promotes Shh signalling	(Schou et al., 2017)
Tgfb1	TGF-β-increases Shh signalling to induced fibrosis.	(Chung and Fu, 2013)
Itgav	Itgav is a target of Hedgehog signalling and can modulate Hedgehog signalling	(Kosinski et al., 2010; Zhou et al., 2013)

Aliahmad, P., Kadavallore, A., de la Torre, B., Kappes, D., Kaye, J., 2011. TOX is required for development of the CD4 T cell lineage gene program. J Immunol 187, 5931-5940.

Andreotti, A.H., Schwartzberg, P.L., Joseph, R.E., Berg, L.J., 2010. T-cell signaling regulated by the Tec family kinase, Itk. Cold Spring Harb Perspect Biol 2, a002287.

Azzam, H.S., DeJarnette, J.B., Huang, K., Emmons, R., Park, C.S., Sommers, C.L., El-Khoury, D., Shores, E.W., Love, P.E., 2001. Fine tuning of TCR signaling by CD5. J Immunol 166, 5464-5472.
Catlett, I.M., Hedrick, S.M., 2005. Suppressor of cytokine signaling 1 is required for the differentiation of CD4+ T cells. Nat Immunol 6, 715-721.
Cevik, S.I., Keskin, N., Belkaya, S., Ozlu, M.I., Deniz, E., Tazebay, U.H., Erman, B., 2012. CD81 interacts with the T cell receptor to suppress signaling. PLoS One 7, e50396.
Chan, L.H., Wang, W., Yeung, W., Deng, Y., Yuan, P., Mak, K.K., 2014. Hedgehog signaling induces osteosarcoma development through Yap1 and H19 overexpression. Oncogene 33, 4857-4866.
Chung, Y., Fu, E., 2013. Crosstalk between Shh and TGF-beta signaling in cyclosporine-enhanced cell proliferation in human gingival fibroblasts. PLoS One 8, e70128.
Collins, S., Lutz, M.A., Zarek, P.E., Anders, R.A., Kersh, G.J., Powell, J.D., 2008. Opposing regulation of T cell function by Egr-1/NAB2 and Egr-2/Egr-3. Eur J Immunol 38, 528-536.
Decker, E.L., Nehmann, N., Kampen, E., Eibel, H., Zipfel, P.F., Skerka, C., 2003. Early growth response proteins (EGR) and nuclear factors of activated T cells (NFAT) form heterodimers and regulate proinflammatory cytokine gene expression. Nucleic Acids Res 31, 911-921.
Hager-Theodorides, A.L., Outram, S.V., Shah, D.K., Sacedon, R., Shrimpton, R.E., Vicente, A., Varas, A., Crompton, T., 2002. Bone morphogenetic protein 2/4 signaling regulates early thymocyte differentiation. J Immunol 169, 5496-5504.
Jones-Mason, M.E., Zhao, X., Kappes, D., Lasorella, A., Iavarone, A., Zhuang, Y., 2012. E protein transcription factors are required for the development of CD4(+) lineage T cells. Immunity 36, 348-361.
Kishimoto, H., Sprent, J., 1997. Negative selection in the thymus includes semimature T cells. J Exp Med 185, 263-271.
Kosinski, C., Stange, D.E., Xu, C., Chan, A.S., Ho, C., Yuen, S.T., Mifflin, R.C., Powell, D.W., Clevers, H., Leung, S.Y., Chen, X., 2010. Indian hedgehog regulates intestinal stem cell fate through epithelial-mesenchymal interactions during development. Gastroenterology 139, 893-903.
Lauritsen, J.P., Kurella, S., Lee, S.Y., Lefebvre, J.M., Rhodes, M., Alberola-Ila, J., Wiest, D.L., 2008. Egr2 is required for Bcl-2 induction during positive selection. J Immunol 181, 7778-7785.
Moran, A.E., Holzapfel, K.L., Xing, Y., Cunningham, N.R., Maltzman, J.S., Punt, J., Hogquist, K.A., 2011. T cell receptor signal strength in Treg and iNKT cell development demonstrated by a novel fluorescent reporter mouse. J Exp Med 208, 1279-1289.
Nowyhed, H.N., Huynh, T.R., Blatchley, A., Wu, R., Thomas, G.D., Hedrick, C.C., 2015. The nuclear receptor nr4a1 controls CD8 T cell development through transcriptional suppression of runx3. Sci Rep 5, 9059.
Outram, S.V., Hager-Theodorides, A.L., Shah, D.K., Rowbotham, N.J., Drakopoulou, E., Ross, S.E., Lanske, B., Dessens, J.T., Crompton, T., 2009. Indian hedgehog (Ihh) both promotes and restricts thymocyte differentiation. Blood 113, 2217-2228.
Outram, S.V., Varas, A., Pepicelli, C.V., Crompton, T., 2000. Hedgehog signaling regulates differentiation from double-negative to double-positive thymocyte. Immunity 13, 187-197.

Development • Supplementary information

Puls, K.L., Hogquist, K.A., Reilly, N., Wright, M.D., 2002. CD53, a thymocyte selection marker whose induction requires a lower affinity TCR-MHC interaction than CD69, but is up-regulated with slower kinetics. Int Immunol 14, 249-258.
Raman, V., Blaeser, F., Ho, N., Engle, D.L., Williams, C.B., Chatila, T.A., 2001. Requirement for Ca2+/calmodulin-dependent kinase type IV/Gr in setting the thymocyte selection threshold. J Immunol 167, 6270-6278.
Roberts, D.J., Johnson, R.L., Burke, A.C., Nelson, C.E., Morgan, B.A., Tabin, C., 1995. Sonic hedgehog is an endodermal signal inducing Bmp-4 and Hox genes during induction and regionalization of the chick hindgut. Development 121, 3163-3174.
Schou, K.B., Mogensen, J.B., Morthorst, S.K., Nielsen, B.S., Aleliunaite, A., Serra-Marques, A., Furstenberg, N., Saunier, S., Bizet, A.A., Veland, I.R., Akhmanova, A., Christensen, S.T., Pedersen, L.B., 2017. KIF13B establishes a CAV1-enriched microdomain at the ciliary transition zone to promote Sonic hedgehog signalling. Nat Commun 8, 14177.
Shao, H., Kono, D.H., Chen, L.Y., Rubin, E.M., Kaye, J., 1997. Induction of the early growth response (Egr) family of transcription factors during thymic selection. J Exp Med 185, 731-744.
Singer, N.G., Fox, D.A., Haqqi, T.M., Beretta, L., Endres, J.S., Prohaska, S., Parnes, J.R., Bromberg, J., Sramkoski, R.M., 2002. CD6: expression during development, apoptosis and selection of human and mouse thymocytes. Int Immunol 14, 585-597.
Steinke, F.C., Yu, S., Zhou, X., He, B., Yang, W., Zhou, B., Kawamoto, H., Zhu, J., Tan, K., Xue, H.H., 2014. TCF-1 and LEF-1 act upstream of Th-POK to promote the CD4(+) T cell fate and interact with Runx3 to silence Cd4 in CD8(+) T cells. Nat Immunol 15, 646-656.
Suzuki, A., Yamaguchi, M.T., Ohteki, T., Sasaki, T., Kaisho, T., Kimura, Y., Yoshida, R., Wakeham, A., Higuchi, T., Fukumoto, M., Tsubata, T., Ohashi, P.S., Koyasu, S., Penninger, J.M., Nakano, T., Mak, T.W., 2001. T cell-specific loss of Pten leads to defects in central and peripheral tolerance. Immunity 14, 523-534.
Zhou, X., Qiu, W., Sathirapongsasuti, J.F., Cho, M.H., Mancini, J.D., Lao, T., Thibault, D.M., Litonjua, A.A., Bakke, P.S., Gulsvik, A., Lomas, D.A., Beaty, T.H., Hersh, C.P., Anderson, C., Geigenmuller, U., Raby, B.A., Rennard, S.I., Perrella, M.A., Choi, A.M., Quackenbush, J., Silverman, E.K., 2013. Gene expression analysis uncovers novel hedgehog interacting protein (HHIP) effects in human bronchial epithelial cells. Genomics 101, 263-272.

Table S2: PCA Gene list with relevant PC scores and Intersected Gene list for each sorted thymocyte population.

Click here to Download Table S2
